# A method to computationally screen for tunable properties of crystalline alloys

**DOI:** 10.1016/j.patter.2023.100823

**Published:** 2023-08-11

**Authors:** Rachel Woods-Robinson, Matthew K. Horton, Kristin A. Persson

(Patterns *4*, 100723; May 12, 2023)

In the originally published version of this article, a sentence in the subsection “Example of screening alloy pairs for p-type transparent conductors” read “The gray p-type TC regime depicts a range of parameter space where 2.9 eV ${<E}_{G}^{PBE}<$ 3 eV (and thus possible experimental gaps greater than 3 eV, since PBE systematically underestimates $E_{G}$) and…,” whereas it should read “The gray p-type TC regime depicts a range of parameter space where 2.9 eV ${<E}_{G}^{PBE,corr}<$ 5.6 eV (and thus likely to be transparent) and….” This revision does not change the discussed interpretation of the results. The sentence has now been corrected online. The authors apologize for any confusion this error may have caused.

