# Supplementary material for: A method to computationally screen for tunable properties of crystalline alloys
Source: Patterns (N Y). 2023 Apr 7;4(5):100723. doi: 10.1016/j.patter.2023.100723 (PMC10201207; doi:10.1016/j.patter.2023.100723)
Supplement: Document S1. Figures S1–S3 [file mmc1.pdf]

Patterns, Volume *4*

Supplemental information

A method to computationally screen for tunable properties of crystalline alloys

Rachel Woods-Robinson, Matthew K. Horton, and Kristin A. Persson

Supplemental Experimental Procedures

Open-source code

All code used in the preparation of this manuscript is open source. Where future developments require changes to the methods or algorithms described in this manuscript, these open source codes will contain the ground truth for how the alloy database is constructed.

The codes developed were:

- `pymatgen-analysis-alloys` An add-on package for the `pymatgen` code that contains the `AlloyPair`, `AlloyMember`, `AlloySystem` and `FormulaAlloyPair` classes and related logic.
- `emmet` This is an existing package containing information on how to build the databases used by the Materials Project. Code was added to `emmet-core` to define the database document schema and `emmet-builders` to define the scripts to construct the database in a scalable manner. Code was added to `emmet-api` to allow researchers to access the alloy database constructed in this work through the Materials Project.

At the time of writing, `pymatgen-analysis-alloys` is installable using the Python Package Index via `pip install pymatgen-analysis-alloys` and importable via `import pymatgen.analysis.alloys`. The main classes are located in `pymatgen.analysis.alloys.core` and are documented and unit tested. Readers are encouraged to refer to the code for any updates to this methodology subsequent to publication.

Unique Identifiers

This work uses a document-based database, namely MongoDB, which does not have an explicit schema. The database fields present will be derived based on the available attributes in the `AlloyPair` and other objects. The canonical reference for these attributes is the code itself.

Nevertheless, the use of a unique, primary key is essential for database management.

For `AlloyPair` this is an underscore-delimited string containing the unique identifiers of the endpoints from whatever input database is used. This implicitly assumes that an underscore is not used in the input databases's unique identifiers. For example, an `AlloyPair` consisting of materials mp-804 (GaN) and mp-661 (AlN) would have the unique identifier "mp-661_mp-804". The `AlloyPair` construction orders the endpoints deterministically, such that AlN will always be endpoint "A" and GaN will always be endpoint "B" regardless of the order of endpoints provided during construction.

For `AlloySystem`, the unique identifier is based on the first six digits of the MD5 hash of a sorted, underscore-delimited list of all unique identifiers of individual materials in that alloy system. This ensures that the identifier will change as additional members are added to the alloy system.

Database Building

Constructing the entire alloy database is CPU-bound and takes approximately one day on a 2.3 GHz 8-core Intel CPU. For Materials Project production purposes, this database build is typically parallelized across multiple nodes and "pleasingly parallel", since it can be parallelized across anonymous formula (for alloy pair and alloy system construction) and across chemical system (for alloy member construction) such that the total build time is greatly reduced.

Statistical analysis of interpolated half-space hull energies

In **Figure S1** we have performed a simple statistical analysis on a set of formula alloy pairs in which there is one or more values of x with at least two members (i.e. polymorphs) belonging to unique alloy pairs with unique endpoints. Rather than compare whether the interpolated energy of a member polymorph i (E_i^{interp}) precisely matches its DFT predicted from MP ($E_i^{\mathrm{hull,MP}}$), we instead have focused on whether the relative energy difference between two polymorphs is comparable (ΔE_{dif}). At the current build of the database, this sample set amounts to 379 values of x across 323 formula alloy pairs, and we perform statistical analysis on the two lowest energy polymorphs for each value of x. With this analysis, we show that approximately 64% of polymorphs are predicted using alloy pair interpolation to be within 25 meV of their MP orderings, and approximately 91% within 100 meV. Therefore a majority of cases are predicted to have the same polymorph ordering as in MP, although there are outliers and it is important to keep this in mind when using the alloy database.

With this statistical analysis we caution that we are not trying to prove a result, but rather provide insight into the distribution of data in the alloy database. The MP data here are formation energies of ordered approximations of alloys; these are not equivalent to the formation energies of the actual alloy (e.g., of a solid solution). MP has not done exhaustive enumerations of orderings, and it is unclear whether a given ordering is a useful approximation or not. We also note that there is bias in terms of which formula alloy pairs have members and which do not, inherited from the inherent bias in the MP database (e.g., oversampling of Li-containing systems due to battery applications; see Figure 4 and discussion), so these trends may not be

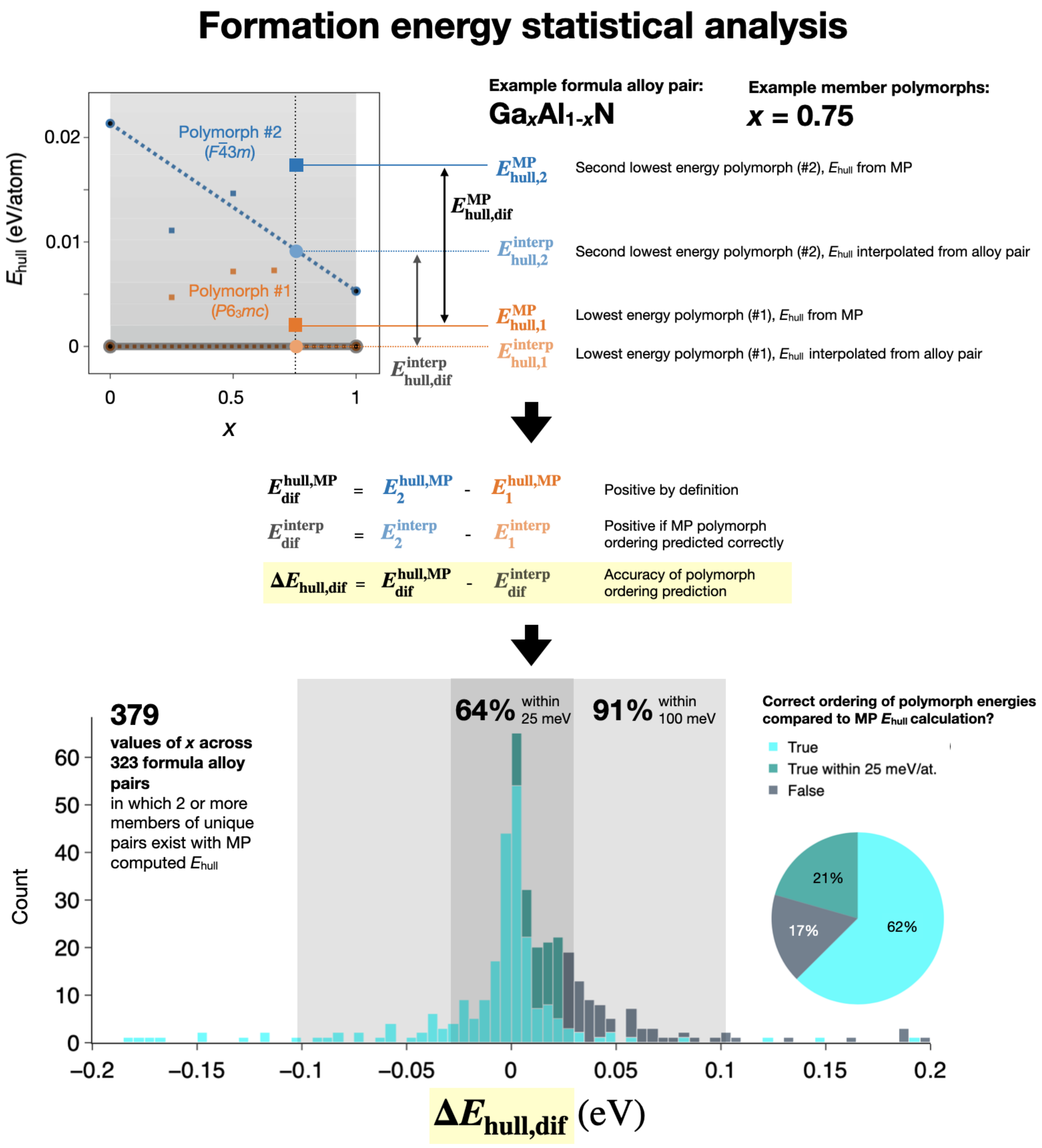

Figure S1: Half-space hull statistical analysis. A simple statistical analysis of interpolated polymorph ordering using the half-space hull framework. For reference, the formula alloy pair for $Ga_xAl_{1-x}N$ is shown (see Figure 4 in the manuscript, focusing on the two lowest-energy polymorphs at x=0.75. Square markers denote MP E_{hull} values, while circular markers denote interpolated values.

representative of all chemistries and structures. The trend here however is that, as the databases increase in size (e.g., more and more ordered approximations of different disordered materials are added), it will become more accurate.

Merging of AlloySystem

Consider there is an alloy system containing an endpoint with anonymous formula ABC. However, this endpoint ABC is also found to be a member of another alloy system (say, a system made up of the two endpoints, AB and AC). In this case, we can conclude the first alloy sys-

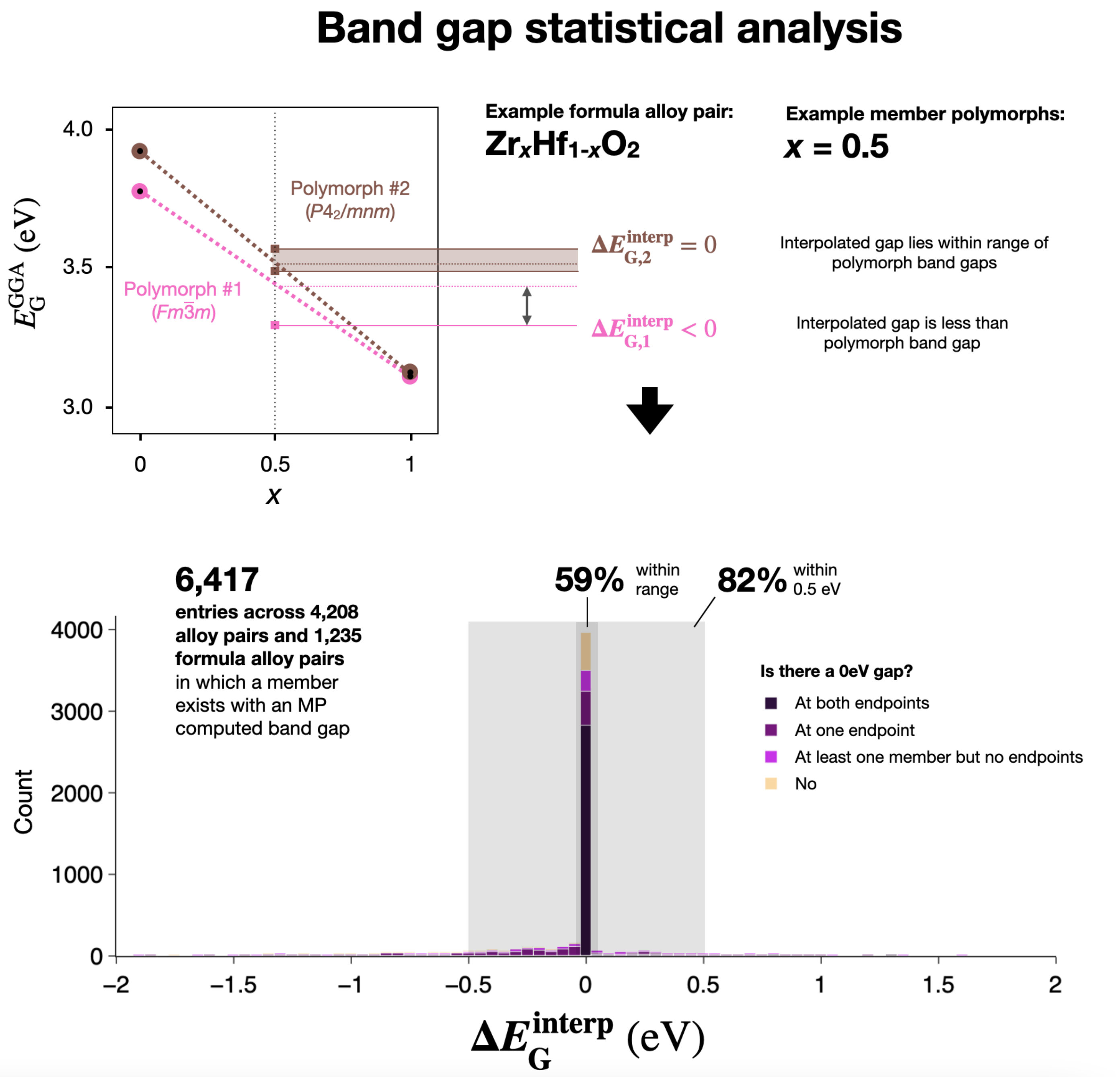

Figure S2: Band gap statistical analysis.A simple statistical analysis of interpolated band gap of alloy pair members, compared to their GGA band gaps on the MP database. For reference, the formula alloy pair for $Zr_xHf_{1\text{-}x}O_2$ is shown (see **??**, focusing on the $P42/nmc$ and $Fm-3m$ polymorphs at x=0.5. Square markers denote MP E_{hull} values, while circular markers denote interpolated values.

tem can be subsumed into the second alloy system. This process of "merging" alloy systems is important to remove spurious alloy systems, but also presents a subtle problem since whether a material should be considered an alloy or, simply, a new stoichiometric compound is open to interpretation. For example, chalcopyrite is typically considered a compound in its own right, but under this lens would be seen as an alloy of two zincblende endpoints. Therefore, alloy system merging has not been performed on the database in this work, but has been fully implemented in the code and can be done manually on an as-needed basis.

Vegard's Law Approximations

In the manuscript, we assume Vegard's law applies with no bowing to construct Figure 5, and for properties a, E_{G}, and inverse effective mass (i.e. $\frac{1}{m_{\text{e}}^*}$ and $\frac{1}{m_{\text{h}}^*}$). This is a crude approximation for the purposes of providing a window for a given alloy in which properties might lie, *not* as a way to accurately estimate properties. Consensus in the literature is that Vegard's law is an approximation rather than a strict "law", but that it is valid as a predictor for lattice parameter for ideal solutions in which lattice parameters

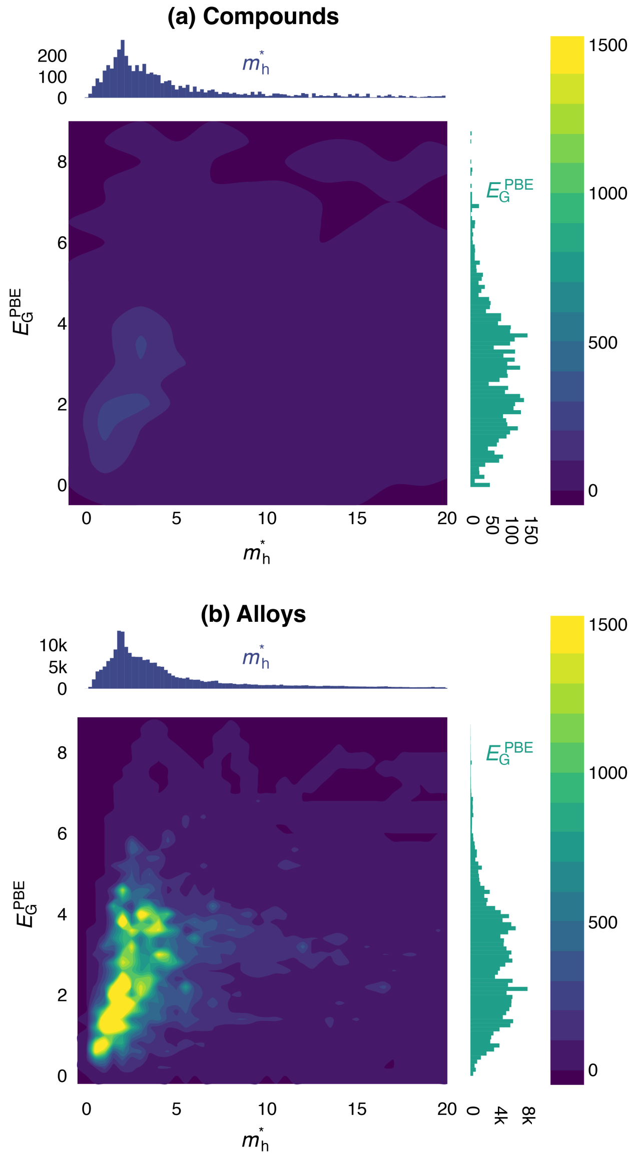

Figure S3: Density plot comparison. 2D density plots showing (a) the distribution of E_G and m_h^* when considering only stoichiometric alloy compounds ("endpoints") and (b) an approximate distribution of E_G and m_h^* including the intermediate alloy compositions, illustrating the different between a discrete and practically continuous distribution of properties.

of the end-points differ by less than 5 %.[1] In **Figure S2** we have performed a simple statistical analysis of Vegard's law interpolated band gaps of alloy pair members, compared to their GGA band gaps on the MP database. At the current build of the database, this sample set amounts to 6,417 entries across 4,208 alloy pairs and 1,235 formula alloy pairs in which a member exists with an MP computed band gap. With this analysis, we show that approximately 59% of interpolated gaps lie within range of MP polymorph band gaps, and approximately 82% lie within 0.5 eV of the MP gap(s).

The literature commonly applies Vegard's law for alloys to estimate a and E_G. In comparison, there is less consensus across the literature about whether Vegard's law is appropriate for m^* and whether bowing is pronounced; this is likely dependent on the specific characteristics of the electronic band structure for the alloy endpoints. According to Piprek[2] and Singh[3], Vegard's law is appropriate for inverse effective mass with the latter providing a derivation. According to Piprek "bowing is not pronounced for the effective mass of most alloys," as compared to stronger bowing for band gap. Here, "most alloys" is likely referring to III-V materials, since these are the dominant class of alloys studied — and for III-Vs, Vegard's law is used in the literature to estimate effective mass e.g. for (AlGaIn)N alloys.[4] Several materials properties, such as the piezoelectric response, are highly nonlinear as a function of alloy content and use of Vegard's law is not appropriate in these cases. Therefore, care must be taken in the choice of which properties associated with a material are included in the database. Moreover, the end user of the database has the ability to incorporate additional properties of the endpoints in whatever manner is most appropriate for their specific property or application.

Alloys increase parameter space

To graphically illustrate how including alloys increases parameter space, **Figure S3** depicts a 2D contour plot of two representative material properties — m_h^* versus PBE E_G (see Methodology) — for (a) compounds in the MP database, i.e. endpoints only, in comparison to (b) candidate alloy materials with steps of $\delta x = 0.01$ in an alloy pair $A_x B_{1-x}$ and assuming Vegard's law with no bowing for E_G and $1/m_h^*$.[3] The histograms above and to the right of each diagram depict the distribution for each individual parameter. Note that this is only an illustration to show the expanded property space accessible when considering alloys, and cannot be not a quantitative comparison since the choice of δ is arbitrary.

[1] Jacob, K., Raj, S., and Rannesh, L. (2007). Vegard's law: a fundamental relation or an approximation? *International Journal of Materials Research*, 98(9):776–779.

[2] Piprek, J. (2013). *Semiconductor optoelectronic devices: introduction to physics and simulation.* Elsevier.

[3] Singh, J. (2007). *Electronic and optoelectronic properties of semiconductor structures.* Cambridge University Press.

[4] Yang, H., Song, T., Liang, X., and Zhao, G. (2015). First-principle study of the electronic band structure and the effective mass of the ternary alloy gaxin1-xp. In *Journal of Physics: Conference Series*, volume 574, page 012048. IOP Publishing.
