# Supplementary material for: A method to computationally screen for tunable properties of crystalline alloys
Source: Patterns (N Y). 2023 Apr 7;4(5):100723. doi: 10.1016/j.patter.2023.100723 (PMC10201207; doi:10.1016/j.patter.2023.100723)
Supplement: Document S2. Article plus supplemental information [file mmc3.pdf]

Patterns

Article

A method to computationally screen for tunable properties of crystalline alloys

Graphical abstract

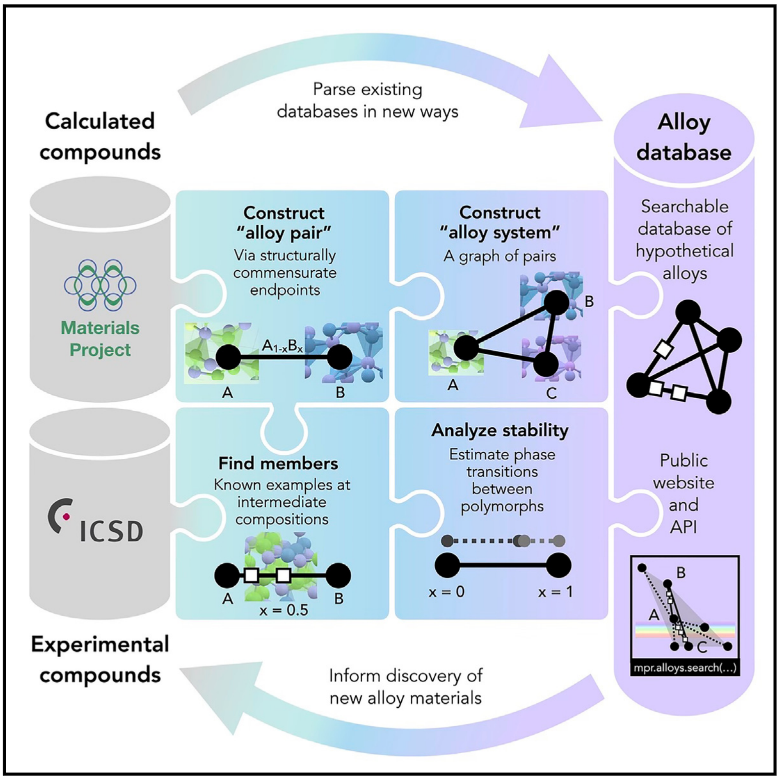

Highlights

- A method is presented to derive potential alloys from existing materials databases
- Use of this database is demonstrated on a screening for a new transparent conductor
- The database is made available via a public Web site and for download via an API
- This work is fully reproducible through use of an accompanying open-source code

Authors

Rachel Woods-Robinson, Matthew K. Horton, Kristin A. Persson

Correspondence

rwoodsrobinson@berkeley.edu

In brief

Typically, computational searches for new materials target stoichiometric "pure" compounds, whereas many real experimental materials are "alloys" or mixtures of existing materials. To address this disconnect, a database of compositionally tunable materials has been created to suggest possible alloys between pairs of already-known materials. This enables discovery of new functional alloy materials that may previously have been overlooked by traditional screenings.

Woods-Robinson et al., 2023, Patterns *4*, 100723
May 12, 2023 © 2023 The Authors.
https://doi.org/10.1016/j.patter.2023.100723

CellPress

Patterns

CellPress
OPEN ACCESS

Article

A method to computationally screen for tunable properties of crystalline alloys

Rachel Woods-Robinson,[1,2,5,6,*] Matthew K. Horton,[2,5] and Kristin A. Persson[3,4]
[1]Applied Science and Technology Graduate Group, University of California at Berkeley, Berkeley, CA 94720, USA
[2]Materials Sciences Division, Lawrence Berkeley National Laboratory, Berkeley, CA 94720, USA
[3]Department of Materials Science and Engineering, University of California at Berkeley, Berkeley, CA 94720, USA
[4]Molecular Foundry Division, Lawrence Berkeley National Laboratory, Berkeley, CA 94720, USA
[5]These authors contributed equally
[6]Lead contact
*Correspondence: rwoodsrobinson@berkeley.edu
https://doi.org/10.1016/j.patter.2023.100723

THE BIGGER PICTURE Ever since the advent of the Materials Genome Initiative, there has been a widespread adoption of high-throughput "materials screenings," whereby a large materials database, typically of many thousands of entries, is queried by a property of interest to suggest candidate materials as the basis for further experimental inquiry. This approach has been applied to discover new materials for varied applications such as batteries, solar panels, and thermoelectrics. However, there exists a disconnect between proposed candidates and device-ready materials: computer-based searches for new materials usually target stoichiometric "pure" compounds, but most real, experimental materials are highly engineered "alloys" or mixtures of compounds. To address this disconnect, in this work a database of tunable materials has been created to suggest possible alloys between pairs of already-known materials. This enables discovery of new materials that may have been entirely overlooked by previous methods.

1 2 3 4 5 **Proof-of-Concept:** Data science output has been formulated, implemented, and tested for one domain/problem

SUMMARY

Conventionally, high-throughput computational materials searches start from an input set of bulk compounds extracted from material databases, but, in contrast, many real functional materials are heavily engineered mixtures of compounds rather than single bulk compounds. We present a framework and open-source code to automatically construct and analyze possible alloys and solid solutions from a set of existing experimental or calculated ordered compounds, without requiring additional metadata except crystal structure. As a demonstration, we apply this framework to all compounds in the Materials Project to create a new, publicly available database of >600,000 unique "alloy pair" entries that can be used to search for materials with tunable properties. We exemplify this approach by searching for transparent conductors and reveal candidates that might have been excluded in a traditional screening. This work lays a foundation from which materials databases can go beyond stoichiometric compounds and approach a more realistic description of compositionally tunable materials.

INTRODUCTION

The power of functional semiconductor materials lies in the tunability of their properties. Since the dawn of the Semiconductor Age, traditional semiconductors—elemental (e.g., Si), IV-IVs (SiC), III-Vs (GaN, GaAs, InGaN), II-IVs (CdTe), etc.—have been manipulated in the laboratory through doping, alloying, processing, and other techniques to yield desired properties. Tunable semiconductor alloy materials enable a variety of energy and optoelectronic applications that govern our modern world, from light-emitting diode (LED) materials, e.g., $In_{1-x}Ga_xN$ (InGaN),[1] to infrared detectors, e.g., $Pb_{1-x}Sn_xTe$ and $Hg_xCd_{1-x}Te$,[2] to piezoelectrics, e.g., $PbZr_xTi_{1-x}O_3$,[3] and are critical for the transformation to renewable energy in solar cell materials; e.g., $CuIn_xGa_{1-x}(S_ySe_{1-y})_2$ (CIGS)[4] and $CdSe_xTe_{1-x}$ (CdTe). The properties of each of these materials reach far beyond those of their

This is an open access article under the CC BY license (http://creativecommons.org/licenses/by/4.0/).

endpoint compositions; e.g., the band gap of InGaN is tunable across a wide range, from ~0.7 eV (InN) to 3.4 eV (GaN). Naturally occurring semiconductor minerals are also stable in alloy forms, e.g., olivine $(Mg_xFe_{1-x})_2SiO_4$, plagioclase $Na_xCa_{1-x}(Al_ySi_{1-y})_4O_8$, and cobaltite $Co_xFe_{1-x}AsS$, indicating a strong tendency toward off-stoichiometric stability.[5]

Meanwhile, in the past decade, computational materials discovery has been advancing novel materials design in a wide range of applications, from thermoelectrics,[6] to Li-ion battery cathodes,[7] to transparent conductors.[8] In most of these cases, materials discovery has been targeted toward stoichiometric "bulk" compounds (also called parent compounds or endpoint compounds in the context of alloys). A candidate compound emerges successfully from a screening if it satisfies a set of property values within a specific cutoff. This methodology has served as a useful starting point, but a grand challenge in the field is determining how to expand this success beyond compounds into off-stoichiometric space to search for ranges of tunability within materials in a high-throughput context. Indeed, a material may be excluded by its endpoint properties without taking into account how its properties can be tuned by doping or alloying. For example, the n-type transparent conductor Sn-doped In_2O_3 is an excellent example of a material where the computed properties of the endpoint compound (In_2 O_3) are not representative of the high experimental performance achieved by introducing tunability.[9] It is recognized that considering all possible off-stoichiometry (defects, dopants, impurity phases and alloying)—intentional as well as unintentional—in the design of novel materials incurs a vast increase in complexity of search space compared with on-stoichiometric compound space. Therefore, part of the challenge is a data problem: how do we manage the additional complexity induced by including off-stoichiometry?

There have been many extensive and notable previous efforts to designing alloys using high-throughput computation. These include but are not limited to the design of high-entropy alloys,[10,11] high-entropy oxides,[12] Heusler compounds,[13] and magnetic Heuslers,[14] as well as alloy design for specific applications including magnetocalorics[15] and thermoelectrics.[16] Such design studies often bootstrap alloy searches from existing computational databases, such as the Materials Project (MP),[17] Automated Flow for Materials Discovery (AFLOW),[18] and The Open Quantum Materials Database (OQMD).[19] Previous efforts have also used novel approaches,[20,21] including machine learning[22] and density functional theory (DFT)-supported calculation of phase diagrams (CALPHAD) methodologies.[23] The importance of considering alloys in high-throughput computation is therefore well known.[24] However, what many of these prior examples have in common is that they are often focused on the generation of new alloy materials within a limited regime of phase space; this is often from the enumeration of possibilities from a single crystal structure prototype or is limited to binary alloys or a restricted chemical space. In contrast, our current work differs in that it offers a general approach for classifying and searching pre-existing high-throughput computational databases. These databases might already contain hidden within them sufficient information to assess the possibility of various alloys existing but require appropriate analysis to unlock. The new analysis capabilities proposed in this work to classify and search existing databases enables more effective materials discovery screenings.

To clarify the scope of this work, we will recap what we mean by alloy in this context. The Hume-Rothery rules,[25] traditionally applied to metals, provide a guideline for considering whether two materials (A and B) may form a substitutional solid solution with each other (A_xB_{1-x}), whereby one atom is replaced by another but the host lattice remains largely unchanged, except for small local distortions. These rules require (1) that the crystal structures of solute and solvent must be similar (that is, commensurate with each other[26]); (2) that the atomic radius of solute and solvent atoms must differ by no more than 15%; (3) that solvent and solute have the same valency for complete solubility; and (4) that the solute and solvent should have similar electronegativity. These rules are good guidelines, although the cutoffs ("15%," "similar") are open to debate. The methodology presented in this work therefore is focused primarily on rule (1) to generate the database using existing algorithms for assessing crystal structure similarity, with sufficient metadata then retained to assess rule (3) by querying the database. Rules (2) and (4) are easily applied by the person retrieving alloys from the database subject to their own materials design requirements; for example, by accessing the database of ionic radii within `pymatgen` to further filter down the list of possible alloys to consider. We emphasize that the alloy database obtained in this work is only a database of *possible* alloys with respect to these rules, and does not guarantee that these alloys do indeed exist. Rather, it is intended as a pre-selection step to guide further inquiry.

Using this database, we create methodology to aid in the analysis of alloying opportunities, enabling computational screening for tunable properties in inorganic alloys when starting from a database of crystallographic structures and associated properties. First, we map tunable material space and search for substitutional alloy compositions and properties within a given set of possible endpoints. Second, we apply this framework to the entire MP database[17] for commensurate[26] (structure matching within a certain tolerance; see section "methodology") structures to enable analysis resulting in over 600,000 potential alloys (alloy pairs), encompassing 270,545 chemical systems and 215 space groups. Third, we provide a series of new techniques to conceptualize and explore this large alloy space, including defining an alloy system composed of alloy pairs, thermodynamic stability estimates of alloys by alloy content using a "half-space hull" approach, and an example of using these data as a pre-selection step in a high-throughput screening. Last, we outline the limitations of this framework and suggest next steps for tunable material screenings.

We focus on semiconductors in this paper, but the general methodology could be applied to any alloy systems where there is a reasonable expectation of structural stability and approximately linearly dependent properties with composition. The alloy framework developed in this work is available in the open-source `pymatgen-analysis-alloys` repository and the analyses and associated enabling functionalities have been incorporated into the MP Web site under a Creative Commons license, with an application programming interface (API) to enable other researchers to explore the data and download the results. These

CellPress
OPEN ACCESS

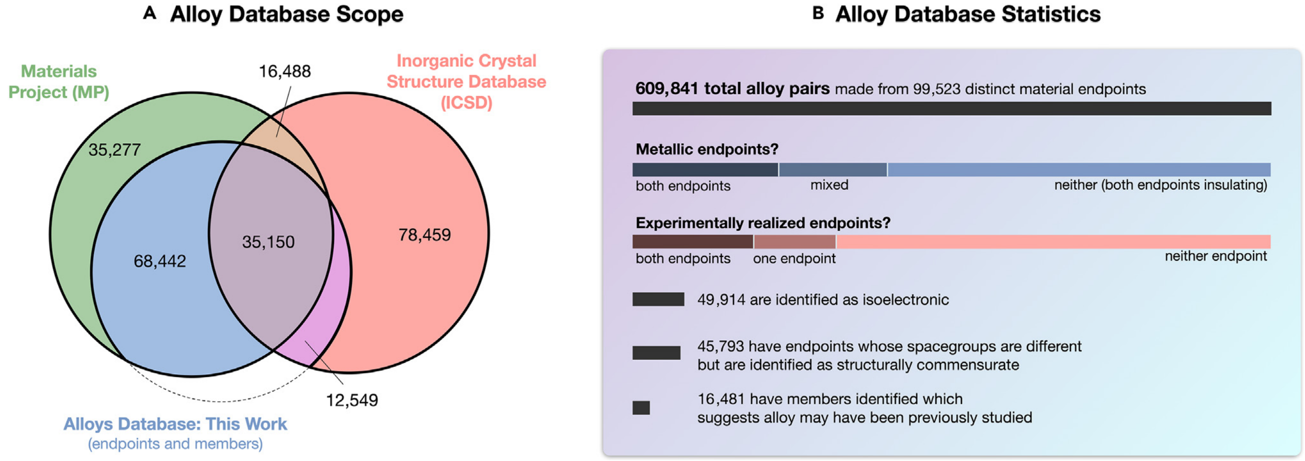

Figure 1. Alloys database overview
(A) A Venn diagram showing materials that are in the Materials Project (MP), associated with an alloy in the alloy database presented in this work (whether an alloy endpoint or an alloy member), and in the Inorganic Crystal Structure Database (ICSD). Note that each entry in MP represents a distinct polymorph, whereas duplicates are present in the ICSD, and so the ICSD is likely overcounted. The Venn diagram gives an overview of how these three databases relate to each other.
(B) A summary of statistics within the alloy database presented in this work.

are online at https://github.com/materialsproject/pymatgen-analysis-alloys and https://materialsproject.org/api respectively.

RESULTS

Creating an alloy database of alloy pairs

In brief, our methods combine sets of structurally commensurate endpoint compounds into an alloy pair database record, which represent two compositions with the possibility of forming a solid solution with one another (see section "methodology" and supplemental information for details). For example, endpoint compounds wurtzite GaN and wurtzite InN form an alloy pair $Al_xGa_{1-x}N$.

Applying the methodology described here to the MP database produces an "alloy database" of 609,841 alloy endpoint pairs and 11,876 alloy systems. Of these candidate alloys, 16,481 pairs (2.7%) and 968 systems (8.1%) are found to contain members of intermediate, non-stoichiometric compositions, suggesting that these may have been previously explored either experimentally or computationally. Figure 1 depicts a summary of the dataset, as a subset of both MP[17] and the Inorganic Crystal Structure Database (ICSD),[27] and is broken down by categories including whether the alloy is metal-metal, metal-semiconductor, or semiconductor-semiconductor, and whether the alloy endpoints have been previously synthesized experimentally. While the candidate alloy pairs are generated from the MP database, alloy members are assigned by searching both the MP and ICSD databases. It is observed that of members from the ICSD, 67% are of disordered compounds, compared to the ICSD as a whole in which 44% are disordered. Note that exact numbers will vary according to the version of the respective database accessed, and reported statistics here reflect the most recent version of the ICSD accessible by MP at the time of publication.

In Figure 1, we also highlight that we have determined 45,793 alloy pairs whose endpoint compounds are not detected to have the same space group. This can either be because the detected space group, being subject to numerical tolerances, is incorrect, or it can be a sign of a phase transition. An instance of the latter case might be one endpoint of an alloy pair having a small polar distortion, while the other endpoint might be a non-polar material; here, the space groups of the endpoints do not match, but the crystal structures might still be sufficiently "commensurate" and able to alloy. This demonstrates the importance of carefully selecting the method for which two materials are considered to be structurally commensurate, and so might form a substitutional alloy. In the context of a materials screening, including alloys drastically expands the accessible and searchable parameter space (see Figure S3 in the supplemental information). When properties of an alloy pair are considered, we take properties of the endpoints when known and assume Vegard's law with no bowing for lattice constant, band gap (E_G) and inverse effective mass ($1/m^*$).[28] We note that excluding bowing is a crude approximation for band gap, but bowing is not as significant for inverse effective mass (see supplemental information).[29]

Exploration of alloy systems

By combining alloy pairs that are all commensurate with one another, alloy systems can be generated (see section "methodology") in which each alloy system spans a region of accessible phase space. Applying this methodology to MP creates a total of 11,876 possible alloy systems. One application of the alloy system framework is the construction of semiconductor bowing plots, which are useful for visualizing lattice matching and band gap tuning in semiconductor alloys and are typically constructed manually via a literature review. A typical example might be a plot showing wurtzite III–V alloys system (GaN, InN, etc.), but this can be generalized for any alloy system. In Figure 2A, we take an example of two systems that have been studied experimentally but not as extensively as the III–V system: zinc-blende II-*Ch* and chalcopyrite I-III-Ch_2 chalcogenide materials.[30] Compounds are grouped by commensurate structure, each marker corresponding to an experimentally observed endpoint

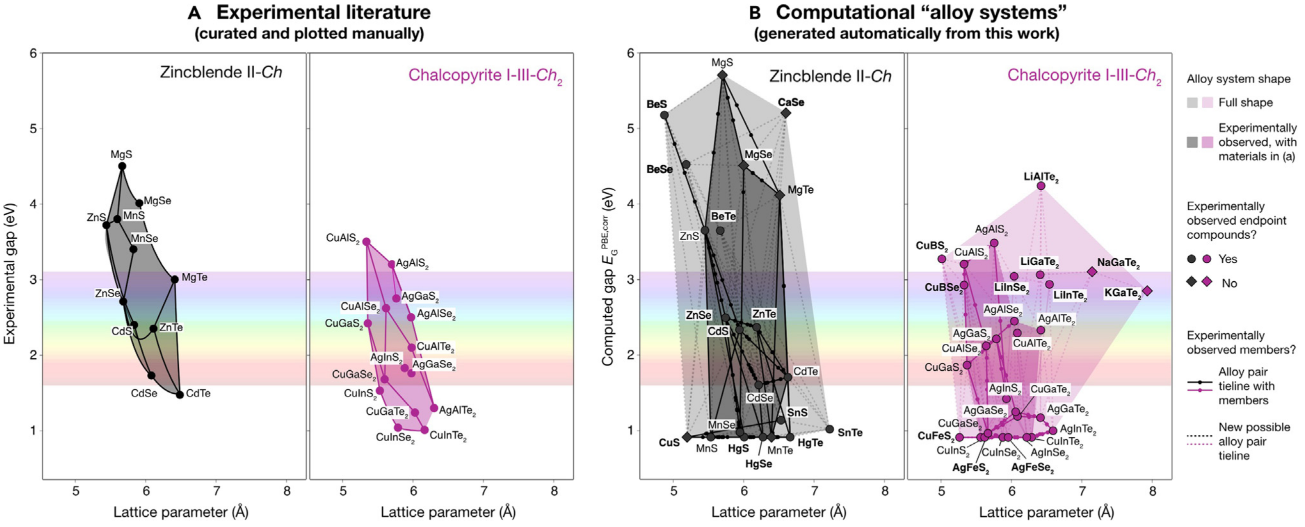

Figure 2. Lattice parameter vs. band gap demo

A comparison of a lattice parameter vs. band gap plot for wide-band-gap zincblende and chalcopyrite chalcogenide materials. (A) A manually constructed plot from the experimental literature,[30] including experimental bowing, and (B) generated computationally from an alloy system in our database. Note that in (B) each alloy system is filtered to include only chalcogenide (S, Se, Te) compounds with commensurate oxidation states, that band gaps $E_G^{PBE,corr}$ are computed PBE gaps with an approximate empirical correction factor,[31] and that the lattice parameter is computed from the conventional unit cell. Boldface in (B) indicates phases not present in (A).

compound, and each line segment corresponds to an experimentally observed alloy (e.g., $Zn_xMg_{1-x}S$). Most of the compounds plotted are in their most stable polymorph; however, we note some exceptions (e.g., MnN, MnSe, and CdSe have a more stable polymorph than zincblende, but zincblende is plotted here for clarity and completeness).

Using the alloys systems framework, we generate corresponding alloy systems for zincblende and chalcopyrite chalcogenide semiconductors. These systems are plotted in the two panels of Figure 2B as a function of lattice parameter and band gap, where each commensurate system is merged into a shape to represent their range. Alloy systems are generated as a function of a single compound—in this case, zincblende ZnS and chalcopyrite $CuAlS_2$—and then outputs are filtered to include only chalcogenide compounds with commensurate oxidation states. Since semi-local DFT underestimates the band gap, we plot Purdue-Berke-Ernzerhof (PBE) gaps with an applied approximate empirical correction factor from the literature, denoted as $E_G^{PBE,corr}$.[31] Discrepancies between the experimental plots and plots derived from the database derive mainly from errors as a result of using the PBE functional. Errors in predicted gap are also exacerbated across the database in magnetic systems where the magnetic order has not been predicted; for example, in Figure 2, it is shown that zincblende MnS (Materials Project: mp-1783) is predicted with PBE to have a band gap of 0 eV but experimentally has been shown to have a gap of approximately 3.8 eV[32]; in this case, it is because the database entry was calculated in a ferromagnetic configuration rather than the correct antiferromagnetic configuration. For better accuracy, we recommend performing additional hybrid functional calculations to complement the initial screening and provide a better estimate the gap in the alloy database or, in the future, using more accurate calculations to construct the database. We emphasize that the purpose of this work is not to demonstrate accurate band gap prediction, since more accurate methods are already well known, but to demonstrate the machinery of constructing alloy pairs and connecting these into alloy systems for the purposes of a materials discovery screening.

We observe in Figure 2 that the shapes and features of computationally generated alloys systems in (b) qualitatively match the experimental diagrams in (a), subject to uncertainties in band gaps as explained above. Additionally, more information is captured in (b); in particular, the members (MP and ICSD) of many of the alloy pairs are denoted to indicate which alloys have seen previous study. Including additional hypothetical alloy pairs here increases the range of search space, by nearly 50% for II-*Ch* and by over 50% for I-III-Ch_2, and new alloy pairs are marked with dotted lines such as $Ca_xCd_{1-x}Se$ and $AgAl(Se_xTe_{1-x})_2$. The computed alloy system plots can also inspire new materials design searches over a variety of multinary alloys. For example, in a search for an amber light-emitting diode (LED) material (~580–590 nm; i.e., 2.10–2.14 eV) with a lattice parameter matched to zincblende GaAs (5.6531 Å[33]), one may examine the region around $Zn_xMn_{1-x}S$ or $CuAl_xGa_{1-x}Se_2$ alloy pairs.

In principle, for a given alloy system, an alloy's lattice parameter (or volume cube root, if comparing non-cubic systems) and band gap can be tuned within the bounds of the shape bounded by the alloy end-members in the plot by varying alloy composition. Here we show a plot for a simple comparison of *a* vs. E_G for conventional semiconductors, but alloy system plots can be created for any set of properties and can in principle be expanded into higher dimensions. Some degree of bowing is likely in these systems, as shown in Figure 2A. Additionally, discontinuities in Vegard's law can arise when gaps transition from direct to indirect nature across alloy space. However, this

CellPress
OPEN ACCESS

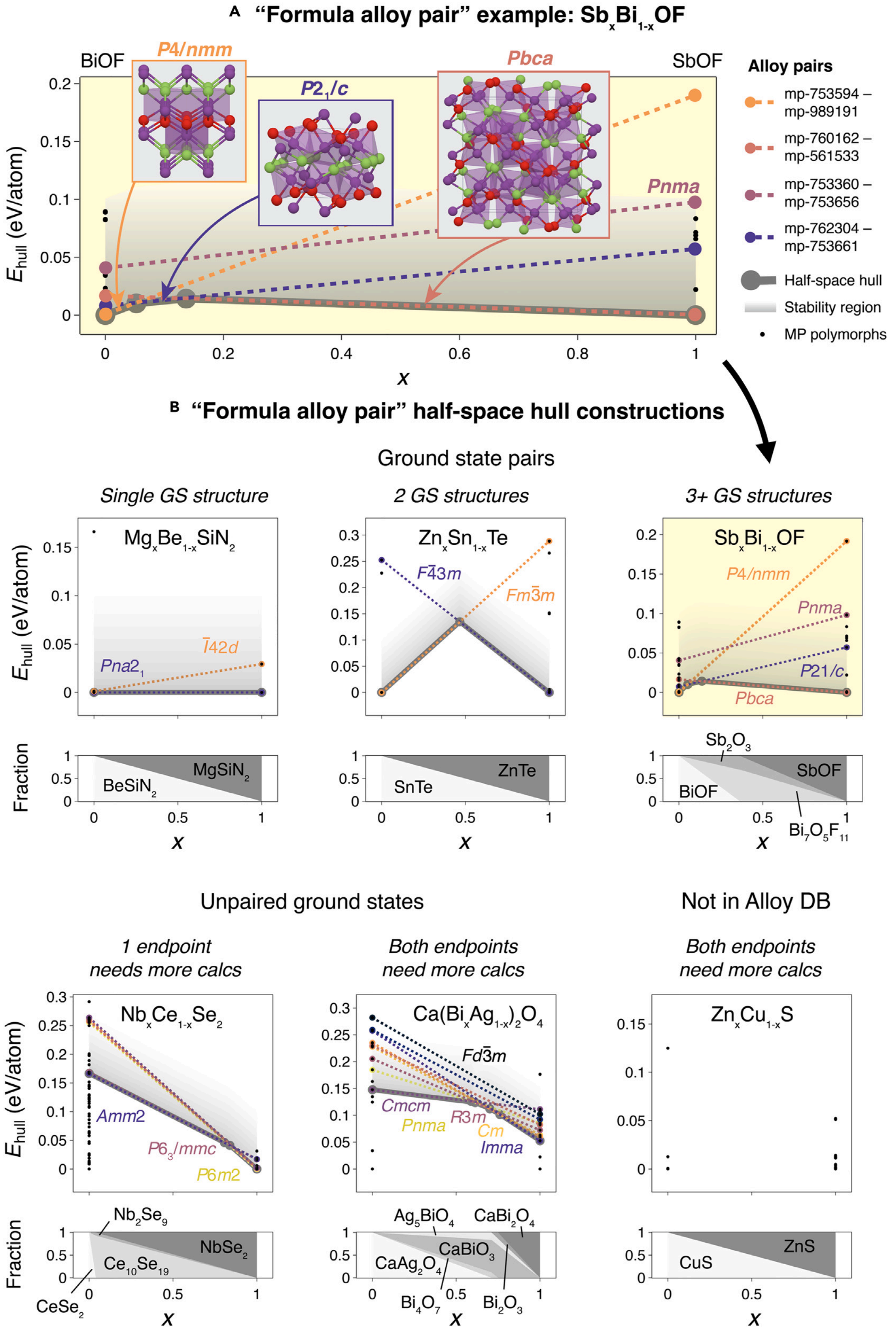

Figure 3. Half-space hull stability analysis

(A) A representative formula alloy pair of $Sb_xBi_{1-x}OF$, with three unique phases lying on the half-space hull. Dotted lines depict where a competing polymorph becomes stable. Decomposition plot on the right shows thermodynamic decomposition products from a ternary phase diagram, as a function of x.

(legend continued on next page)

analysis is helpful as an initial guide and provides a systematic method for generation of such figures, which are already commonplace in the literature.

Estimating alloy stability

So far, we have introduced a set of alloys that are purely hypothetical, yet have not discussed thermodynamic stability and synthesizability. Computationally determining synthesizability of a given alloy is non-trivial and requires constructing a temperature-dependent phase diagram, computing the effects of various entropy terms, and potentially considering the effects of nucleation and kinetics. A simple (yet imperfect, due to the typical T = 0K approximation of DFT) metric to assess stability in solid state compound materials is the energy above the convex hull (E_{hull}), where an E_{hull} of 0 eV/atom defines a thermodynamic ground state, and E_{hull} is reported for compounds in the MP database as derived from phase diagrams constructed from DFT calculations. For alloys, even determining this simple E_{hull} metric is non-trivial; it requires computing a variety of orderings, as well as fully exploring possible competing polymorph phases and all of their possible orderings (see section "discussion").

To approximate whether a given alloy may be stable or synthesizable, and as a first step before performing additional in-depth calculations, we have calculated a hull across alloy content using the half-space intersection of the lines representing the linear interpolation of formation enthalpies between the two alloy endpoints—hereafter called a "half-space hull"—to identify ranges of alloy content x at which different polymorphs might be stable. This is defined by a "formula alloy pair" and "alloy segments" (see section "methodology"). To use this database effectively, the user should make sure to consider entropic terms. Some of these, such as configurational entropy, are trivial to calculate under certain assumptions, while others, such as vibrational or electronic entropy, might require further calculations or more sophisticated models.

Figure 3 depicts a set of formula alloy pair diagrams derived from the alloy database, made up of alloy pairs that all have the same composition (A_xB_{1-x}). For example, a $Sb_xBi_{1-x}OF$ formula alloy pair is magnified in Figure 3A and shown in the third panel of (b), with BiOF endpoint compounds on the left side (x = 0) and SbOF endpoint compounds on the right side (x = 1), and with the y axis representing E_{hull}. Only compounds that are present in the MP database are included here. For each case where a BiOF compound is structurally commensurate with a SbOF compound, an `AlloyPair` is formed and a colored dotted line is drawn in Figure 3A. For example, $P4/nmm$ BiOF (Materials Project: mp-753594, on the hull) is connected with $P4/nmm$ SbOF (Materials Project: mp-989191, with E_{hull} = 0.192 eV) by a blue dotted line, while $Pcba$ BiOF (Materials Project: mp-760162, with E_{hull} = 0.017 eV) is connected with $P4/nmm$ SbOF (Materials Project: mp-561533, on the hull) by a green dotted line. In this formula alloy pair, $P2_1/c$ (purple) and $Pnma$ (red) pairs are also drawn.

The half-space hull is drawn as a continuous gray line in Figure 3A. The changes of slope along this line represent alloy segments (see section "methodology"), which represent phase changes as x is increased. Thus, in this example the half-space hull defines segments of $Sb_xBi_{1-x}OF$ where $P4/nmm$ is the lowest energy phase ($0 \leq x \lesssim 0.05$), where $P2_1/c$ is the lowest energy phase ($0.05 \lesssim x \lesssim 0.15$), and where $Pbca$ is the lowest energy phase ($0.15 \lesssim x \leq 1$). Since a phase does not have to lie on the hull to be synthesizable, we draw a region above the half-space hull (the stability region in a shaded gray gradient) at which the energy above the half-space hull is less than 0.1 eV/atom. It is typical in materials screenings to define an arbitrary cutoff such as this, below which materials are more likely to be synthesizable. While this choice of cutoff is reasonable for many semiconductors, and especially oxides,[34] we note that it would likely be far smaller for metallic alloys[35] and may be larger for nitrides and other non-oxide semiconductors.[36] The choice of cutoff is a free parameter for the user of this database, and we encourage users to carefully consider which cutoff is most appropriate for their application.

According to the selected cutoff of 0.1 eV/atom used in Figure 3, it may be possible to synthesize alloys that lie within the gray region, rather than only the alloys that lie directly upon the half-space hull. For example, it may be possible to synthesize $Pbca$ $Sb_xBi_{1-x}OF$ at small values of x, where it is not the lowest energy polymorph, because the linearly interpolated energy is still below this cutoff and close to that of competing polymorphs. However, it is far less likely that $P4/nmm$ $Sb_xBi_{1-x}OF$ solid solutions could be synthesized at high values of x, since the interpolated energy for this alloy pair lies well outside of the 0.1 eV/atom stability region. We note that there are other endpoint compounds that do not have commensurate pairs (black circular markers), and, for this method to be technically complete, the formation energies of the commensurate structure pairs for these polymorphs would have to be computed.

Figure 3B depicts other possible scenarios of formula alloy pairs within the alloys database. Here, a paired ground state implies that, for the ground state of a given endpoint, a commensurate structure at the composition of the other endpoint exists, forming an alloy pair. An unpaired ground state implies that the ground state structure of one endpoint does not have a commensurate structure at the composition of the other endpoint, and this is an indication to us that more calculations should be performed in order to complete the formula alloy pair. Cases where both endpoints have paired ground states (three examples on the left) are most likely to provide useful information using the half-space hull method. For example, in $Mg_xBe_{1-x}SiN_2$, both ground states are $Pna2_1$, and thus it is likely that a solid solution can be synthesized across all values of x with this structure retained. In $Zn_xSn_{1-x}Te$, both endpoints have commensurate ground states and no other known polymorphs with $E_{\mathrm{hull}} < 0.1$ eV/atom. Thus, a phase change from $Fm\bar{3}m$ to $F\bar{4}3m$ is expected at approximately x = 0.5 using the half-space hull formalism. However, there are systems where one or both of

(B) A set of half-space hull intersections, a simple interpolation based on endpoint formation energies to find cross-overs, for six representative formula alloy pairs. Paired means that commensurate structures exist, while unpaired means that no commensurate structure is known in the database. It is not expected that these cross-over points will be exact, but they might provide an estimate. For each alloy system, this then gives a range of allowed compositions and phases. Below each half-space hull construction, a decomposition diagram is plotted as a function of x.

CellPress
OPEN ACCESS

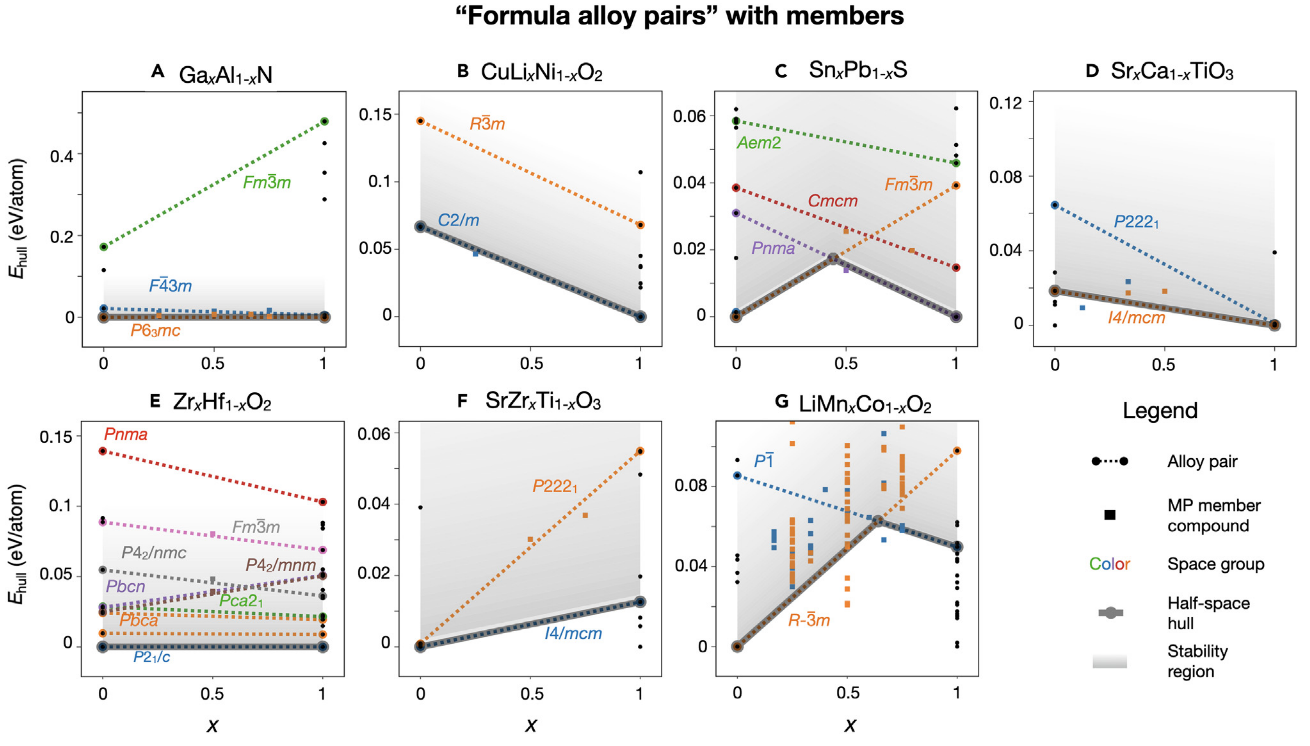

Figure 4. Formula alloy pairs with members

Examples of seven representative formula alloy pairs with members included. The E_{hull} of each member is sourced from the MP database, and can be compared with the linearly interpolated formation energy for each alloy pair. Alloy pairs (dashed lines) and members of alloys pairs (square markers) are colored by space group, and plots are as described in Figure 3.

the ground states do not have a commensurate pair (three examples on the right), such as $Nb_xCe_{1-x}Se_2$ and $Ca(Bi_xAg_{1-x})_2O_4$, and thus more calculations are needed in order to construct a reliable half-space hull. We note that this would add more possibly unstable or unsynthesizable endpoints.

Below each formula alloy pair in (a) is a fractional decomposition diagram. This consists of the various thermodynamic decomposition products and their fractional ratio, as a function of *x*, and is computed using existing functionality in `pymatgen`. The decomposition products give an indicator of possible competing phases that may impede the formation of a solid solution; for example, if a well-known material exists as a possible decomposition product, it is less likely the alloy might be synthesizable. The decompositions products are derived from the full phase diagram of the appropriate chemical system. In $Mg_xBe_{1-x}SiN_2$ and $Zn_xSn_{1-x}Te$, the decomposition products consist solely of the endpoint compounds, and their relative fractional ratio changes monotonically with *x*. However the fractional decomposition of $Sb_xBi_{1-x}OF$, enlarged and plotted on the right-hand panel of (b), is more complicated and consists of four decomposition products: endpoints BiOF and SbOF, as well as Sb_2O_3 and $Bi_7O_5F_{11}$. Thus, although the half-space hull interpolated energies lie below 0.1 eV/atom, these $Sb_xBi_{1-x}OF$ alloys may be challenging to synthesize due to competing thermodynamic reaction products.

As a check to whether the half-space hull is appropriate as a screening tool—or, in other words, whether the linearly interpolated half-space hull estimate is consistent with the DFT computed convex hull of known alloy members—we can include members on these plots for systems in which members are present in databases and their E_{hull} values are known. For example, in Figure 4A we showcase members in the formula alloy pair construction for $Ga_xAl_{1-x}N$. It is shown that the calculated formation enthalpy of the wurtzite (space group $P6_3mc$) alloy members lie below the zincblende (space group $F\bar{4}3m$), which is consistent with the half-space hull; here, these data points refer to the formation enthalpies as calculated with DFT using small ordered approximations from entries already existing in the MP database. In Figures 4B–4G, we plot six other examples of formula alloy pair half-space hull constructions for which there are members included in the alloy pairs. For $Zr_xHf_{1-x}O_2$ (e), the calculated formation energies for space groups $P4_2/mnm$, $P4_2/nmc$, and $Fm\bar{3}m$ at $x = 0.5$ lie nearly exactly on the linearly interpolated energies. Other systems (e.g., $Sr_xCa_{1-x}TiO_3$ and $Sn_xPb_{1-x}S$) have alloys ranked in the same order as the half-space hull prediction, albeit not precisely on the predicted lines. We note that some alloys are extensively sampled in the MP database such as $LiMn_xCo_{1-x}O_2$, likely due to its interest as a battery material leading to a large amount of calculations performed on this compound with varying degrees of lithiation. All of these plots are generated using the tools provided by `pymatgen-analysis-alloys` and can be similarly constructed for any system of interest. We also perform a simple statistical analysis on the full set of formula alloy pairs with members, and find this framework yields correct polymorph orderings for a majority of the set (to within 25 meV/atom error for 64% of set, and to within 100 meV/atom error

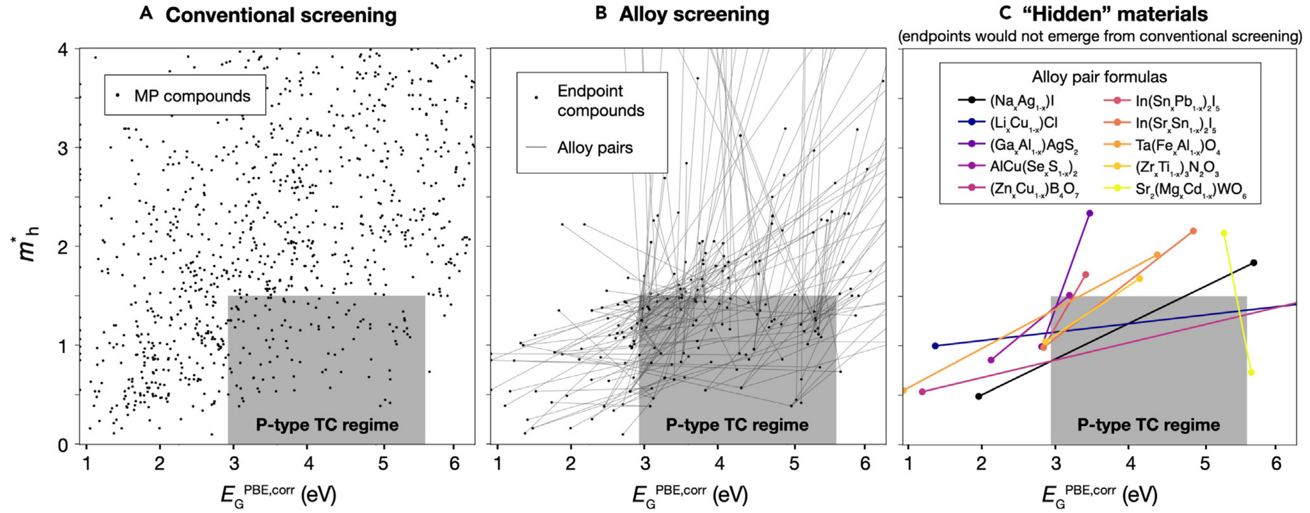

Figure 5. Example screening for p-type TCs

An example of a computational screening of an alloy search space, with the approximate computed p-type TC regime designated with a gray box.

(A) All bulk compounds that intersect the approximate p-type TC regime.

(B) All alloy pairs that intersect the approximate computed p-type TC regime.

(C) Hidden alloy pairs that intersect the p-type TC regime where both endpoints lie outside of the regime. Pairs are denoted by the range of their fractional alloy compositions that lie within the regime, with details denoted in Table 1.

for 91% of the set; see Figure S1 in the supplemental information).

Therefore, when analyzing the set of alloy pairs or alloy systems within our database, it is important to assess which half-space hull scenario a given formula alloy pair lies within and whether there exists a region of phase space where stabilization is likely. Additionally, assessing possible decomposition products informs whether to expect multiple decomposition products, which could impede formation of the alloy. Overall, the half-space hull framework of drawing lines to estimate segments of phase stability is not rigorous, since formation enthalpy does not follow Vegard's law and configurational entropy is not taken into account. Rather, this method is intended to provide an estimate of what alloys might be present and where in alloy space they might be, as a tool to justify or prioritize additional calculations in a high-throughput context, and it is therefore an entrypoint for determining which alloys may be experimentally realizable.

Example of screening alloy pairs for p-type transparent conductors

Including alloys can expand the number of material candidates generated by high-throughput screenings and reveal candidates that otherwise would not have emerged. Here, to demonstrate this quantitatively, we screen our candidate alloy pair dataset for possible p-type transparent conductor (TC) candidates. Discovery of a high-performance p-type TC could enable breakthroughs in solar cells and transparent electronics, among other applications, but to date there are no p-type TCs that perform as well as n-type TCs.[37] A high-performing p-type TC is likely to require a low hole effective mass (m_h^*) to enable high hole mobility and a wide band gap (E_G) to enable optical transparency, among other properties.[9] So far, several data-driven explorations have been performed to search for p-type TC candidates,[8,38,39] but, to our knowledge, no screenings have been performed looking specifically for alloys or specifically for tunable materials rather than compounds.

For this analysis, we use a set of stable or metastable (E_{hull}< 0.1 eV) representative compounds (i.e., alloy endpoints) where m_h^* has been calculated (the same set shown in Figure S3).[40] First, Figure 5A shows a set of bulk compounds from the MP database, with empirically corrected band gap[31] $E_G^{PBE,corr}$ on the x axis and m_h^* on the y axis. The gray p-type TC regime depicts a range of parameter space where 2.9 eV <E_G^{PBE}< 3 eV (and thus possible experimental gaps greater than 3 eV, since PBE systematically underestimates E_G) and m_h^*<1.5, where p-type TC candidates may reside.[9] This figure plots approximately 1,000 compounds, approximately 150 of which lie within the p-type TC region and contain compounds that have emerged from previous screenings (e.g., ZrOS, $TaCu_3S_4$, and Al_2ZnTe_4). The choice of cutoff value tends to be motivated by expected values of physical parameters (e.g., absorption edge and hole mobility), but incur uncertainties in calculated value and inconsistencies between descriptor value and real physical value. Hence, the goal is to suggest a list of target candidates that may be suitable to prioritize for future computational study and experimental inquiry. Therefore, Figure 5A represents a conventional materials discovery screening.

In contrast, Figure 5B depicts a subset of alloy endpoint compounds (black circular markers) and corresponding linearly interpolated alloy pair properties assuming Vegard's law (thin lines between points). This analysis yields 233 alloy pairs whose lines intersect the p-type TC regime, and a subset of 192 alloy pairs in which one or more endpoint lies outside the regime are plotted here for readability. Thus, this plot demonstrates a set of possible, additional alloy pairs to consider as p-type TCs that previously may have been overlooked. The alloy pairs present within the gray region indicate there may be combinations of

CellPress
OPEN ACCESS

Table 1. Hidden alloy pairs with properties of interest to p-type TCs

Pair IDs (A–B)	Alloy formula	Space group	*x* range	E_G^{PBE} range (eV)	m_h^* range	E_{hull}^A (eV/at.)	E_{hull}^B (eV/at.)	On half-space hull?[a]	# decomp. products[b]
mp-22919–mp-23268	$(Na_xAg_{1-x})I$	$Fm\bar{3}m$	0.27–0.74	1.52–2.85	0.85–1.49	0.093	0.000	yes	0
mp-571386–mp-22905	$(Li_xCu_{1-x})Cl$	$Fm\bar{3}m$	0.20–0.52	1.51–3.46	1.13–1.36	0.178	0.020	no	1
mp-684712–mp-32891	$(Y_xGd_{1-x})_2S_3$	$I\bar{4}2d$	0.54–0.57	1.50–1.53	1.49–1.50	0.022	0.036	no	0
mp-5782–mp-556916	$(Ga_xAl_{1-x})AgS_2$	$I\bar{4}2d$	0.62–0.81	1.50–1.59	1.24–1.50	0.000	0.003	yes	0
mp-4979–mp-8016	$AlCu(Se_xS_{1-x})_2$	$I\bar{4}2d$	0.02–0.24	1.50–1.68	1.35–1.50	0.000	0.000	yes	0
mp-756317–mp-3536	$Al_2(Mg_xHg_{1-x})O_4$	$P4/mbm$	0.05–0.16	1.53–1.94	1.21–1.49	0.087	0.000	yes	1
mp-756317–mp-2908	$Al_2(Zn_xHg_{1-x})O_4$	$P4/mbm$	0.07–0.62	1.52–2.91	1.13–1.50	0.087	0.000	yes	1
mp-9081–mp-11742	$CsNd(Te_xS_{1-x})_2$	$R\bar{3}m$	0.55–0.75	1.50–1.67	1.36–1.49	0.002	0.000	yes	0
mp-555093–mp-558690	$(Zn_xCu_{1-x})B_4O_7$	$Cmcm$	0.26–0.63	1.53–3.48	0.85–1.31	0.047	0.058	yes	1
mp-13973–mp-7233	$(La_xGd_{1-x})_2SeO_2$	$P\bar{3}m1$	0.14–0.15	1.50–1.51	1.50–1.50	0.000	0.000	yes	0
mp-23520–mp-23417	$In(Sn_xPb_{1-x})_2I_5$	$I4/mcm$	0.31–0.83	1.50–1.73	1.11–1.49	0.056	0.023	yes	2
mp-23417–mp-23504	$In(Sr_xSn_{1-x})_2I_5$	$I4/mcm$	0.05–0.44	1.50–2.09	1.04–1.50	0.023	0.046	yes	2
mp-754818–mp-756933	$(Tl_xNa_{1-x})TaO_3$	$P4/mbm$	0.36–0.53	1.50–1.91	1.39–1.50	0.087	0.002	yes	0
mp-7482–mp-8402	$Rb(Mg_xHg_{1-x})F_3$	$Pm\bar{3}m$	0.14–0.16	1.52–1.64	1.41–1.47	0.000	0.002	yes	0
mp-760396–mp-761390	$Ta(Fe_xAl_{1-x})O_4$	$I4_1md$	0.31–0.42	1.51–1.79	1.35–1.50	0.056	0.019	no	0
mp-755054–mp-755998	$(Zr_xTi_{1-x})_3N_2O_3$	$Cmcm$	0.06–0.71	1.51–2.13	1.08–1.50	0.008	0.002	no	4
mp-760655–mp-757905	$Li_3(Ti_xBi_{1-x})(PO_4)_2$	$C2/m$	0.44–0.60	1.51–2.08	1.16–1.48	0.066	0.072	yes	6
mp-18903–mp-18848	$Sr_2(Mg_xCd_{1-x})WO_6$	$Fm\bar{3}m$	0.16–0.54	3.39–3.50	0.95–1.50	0.082	0.009	no	0
mp-18848–mp-19400	$Sr_2(Ni_xMg_{1-x})WO_6$	$Fm\bar{3}m$	0.5–0.53	1.52–1.65	1.45–1.50	0.009	0.010	no	0

[a]Whether a composition within *x* range lies on the half-space hull.
[b]Number of decomposition products from half-space hull; excludes endpoint compounds from count.

$E_G^{PBE,corr}$ and m_h^* beyond those represented by the endpoint compounds in Figure 5A.

In Figure 5C, we take this a step further by highlighting a subset of 10 "hidden" alloy pairs that intersect this p-type TC regime but where both of the endpoints lie outside of the regime. This analysis illustrates compounds that themselves are not p-type TC candidates but whose alloys may warrant further exploration. Table 1 reports all the hidden pairs from this analysis, including the 10 hidden pairs from Figure 5C. Included in this table the range of *x* where properties lie within the p-type TC regime (*x* range), the range of $E_G^{PBE,corr}$ and m_h^* achieved within this window, and E_{hull} of the endpoints (where E_{hull}^A corresponds to the first compound of a pair and E_{hull}^B to the second). It is also denoted whether a region of the *x* range lies on the half-space hull, and the number of decomposition products (excluding the endpoint compounds from the count). Most of the alloy pairs that emerge from this screening are quaternaries (alloys of two ternary compounds; e.g., $AlCuS_xSe_{1-x}$), with several ternaries (alloys of binary compounds; e.g., $Cu_xLi_{1-x}Cl$) and quinternaries (alloys of quaternary compounds; e.g., $Sr_2Mg_xCd_{1-x}WO_6$). To our knowledge, none of these alloy pairs have been studied previously as p-type TCs, with the exception of La_2SeO_2 and Gd_2SeO_2, which have been predicted previously using a high-throughput approach.[38] We note that this is just one example of an application where including alloying could yield new material candidates.

DISCUSSION

We have demonstrated a framework to propose new alloys and access the potential tunability of materials for high-throughput screenings. In our presented database, we designate alloy pairs between commensurate endpoint structures; although we present 600,000 unique pairs, this database comprises a subset of possible physical alloys. Several extensions of the presented alloy database are possible, beyond constructing structure-matched pairs. For example, in many experimentally observed alloy systems, endpoints may not structure match within the tolerances we use here but are still commensurate with one another; i.e., they can be connected through a displacive phase transformation (e.g., orthorhombic SnS and rocksalt CaS).[26] Such pairs are not included in this database; however, advances in methodologies for determining whether displacive phase transformations are possible between a given pair of materials could allow the database to be expanded in future.[41,42] In some cases, incommensurate structures, where symmetries are distinct from one another but can be connected through a reconstructive transformation, can also form heterostructural alloys, which are of increased interest for materials design (e.g., rocksalt MnO and wurtzite ZnO can alloy to form $Mn_xZn_{1-x}O$).[26] Similarly, a material might be tuned by varying vacancy concentration topotactically (e.g., NiO_x). Furthermore, there are alloy pairs and alloy systems that in principle could alloy, but have no commensurate endpoint structures currently on MP (e.g., formula alloy pairs labeled "unpaired ground states" and "not in DB" in Figure 3), so, in these systems, more calculations would be required before the alloy could be defined. Nevertheless, in principle, the methodology presented here could be expanded upon to include and categorize all plausible commensurate and incommensurate alloy pairs, and each of the cases mentioned here could be incorporated into future iterations of this alloys database.

We note that the underlying input database from which our alloy database is derived can contain biases. These biases, e.g., concerning structural as well as chemical coverage, can propagate into the alloy database, which should be acknowledged when interpreting results. For example, the MP database necessarily contains many materials that might alloy with each other due to its use of specific structure prediction[43,44] methodologies. As the underlying database expands, this infrastructure has been established to automatically "build" new versions of the alloy database as new data becomes available. Importantly, as better methods for calculating more accurate lattice parameters or band gaps become accessible for high-throughput computation, the alloy database will also incorporate these improved data. The continued building of new versions of this database is an essential aspect of this work, since static datasets have limited utility given the pace of improvement of computational materials databases.

Once a set of potential alloys are suggested from this database, more reliable methods to assess alloy solubility can be used to either rule out or confirm a potential alloy; for example, automated cluster expansions[45,46] or the generalized quasi-chemical approximation (GQCA) method.[47] Our work is intended to serve as a starting point from which to determine systems to consider for such in-depth analyses. The half-space hull diagrams provide a guide to select alloys within a given chemical space, which may be stable and synthesizable. For example, the following calculations of increasing computational cost could be explored based on outputs from the alloys database.

- For compounds at endpoint A (or B) in which a commensurate compound at endpoint B (or A) is not present on MP, there is insufficient information in the database to calculate an alloy pair (for example, the black circular markers in Figure 3 without any connecting line) such as $Zn_xCu_{1-x}S$). Here, the missing compound(s) can be calculated and added to the database. This is still important even if such a compound is unstable or not experimentally realizable at the endpoint, since there may be a region within alloy space where synthesizability becomes possible.
- For alloy pairs in which member compounds are not yet known to exist, members can be calculated (e.g., at x = 0.5) for a few different orderings to assess realizability, or give an indication of expected bowing and other parameters.
- Many real alloy materials are *disordered*, rather than ordered. For members within in alloy pair, special quasi-random structure (SQS) calculations can approximate structures of fully random alloy polymorphs to provide a counterpoint to the small-cell ordered structures more typical in a database such as MP.[48]
- To account for configurational entropy and thermodynamics of specific alloy members, the GQCA can be used to estimate free energy,[47] and subsequently higher-order methods such as cluster expansions can be applied to further investigate specific systems for which high quality phase diagrams are required.[45]

For immediate use, our alloy database has been incorporated into the MP as an app in the new Web site release and API, in the hope that this will serve as a guide for researchers performing screenings of tunable materials. The alloys database will be updated alongside the MP database. A flowchart of the alloys database pipeline and incorporation onto the MP is shown in Figure 6.

Conclusions

In this paper, we have presented a new framework to analyze alloys in the context of materials databases, implemented it into the open-source `pymatgen-analysis-alloys` package, and created an open-source alloys database that has been incorporated into the MP Web site. We have presented a few case studies here of how this database can be utilized in the context of materials research and design.

Importantly, all the analysis presented here has been performed without any new calculations, which showcases some of the data analysis opportunities from mining existing databases. A decade into the Materials Genome Initiative, the materials discovery community has produced large quantities of data in multiple databases, but data *production* is just the start; it is essential that data are curated, structured, and connected in a way to yield the maximum value to the community.

In particular, one of the key challenges is how to link and apply these data to successfully use computational predictions to inform experimental results, especially as experimental databases grow.[49,50] In particular, experimental progress in semiconductors typically starts from a well-studied, well-characterized material and modifies its properties iteratively with the addition of dopants or alloying elements during growth. The framework of this paper addresses this aspect of materials design by creating a database of candidate, tunable materials by a data-focused approach which can use existing materials databases to suggest alloys between pairs of already-known materials. Thus, a new materials screening procedure is now possible that can emphasize experimentally accessible materials and suggest screening outputs that would have been previously wholly overlooked.

EXPERIMENTAL PROCEDURES

Resource availability

Lead contact

Requests for information and resources should be directed to the lead contact, Dr. Rachel Woods-Robinson (rwoodsrobinson@berkeley.edu).

Materials availability

This study did not generate any chemical reagents.

Data and code availability

The alloy framework developed in this work is available in the open-source `pymatgen-analysis-alloys` repository, and the analyses and associated enabling functionalities have been incorporated into the MP Web site under a Creative Commons license, with an API to enable other researchers to explore the data and download the results (see Video S1, Exploring alloys on the Materials Project.). These resources are available online at https://github.com/materialsproject/pymatgen-analysis-alloys and https://materialsproject.org/api respectively. In addition, a static snapshot of the latest version of the database at the time of publication has also been made available at Figshare: 10.6084/m9.figshare.22491793. Note that the database presented in this work is a living resource and will be updated and revised over time to include additional data and fixes where applicable, so data retrieval via the API for any follow-up research purposes is strongly recommended.

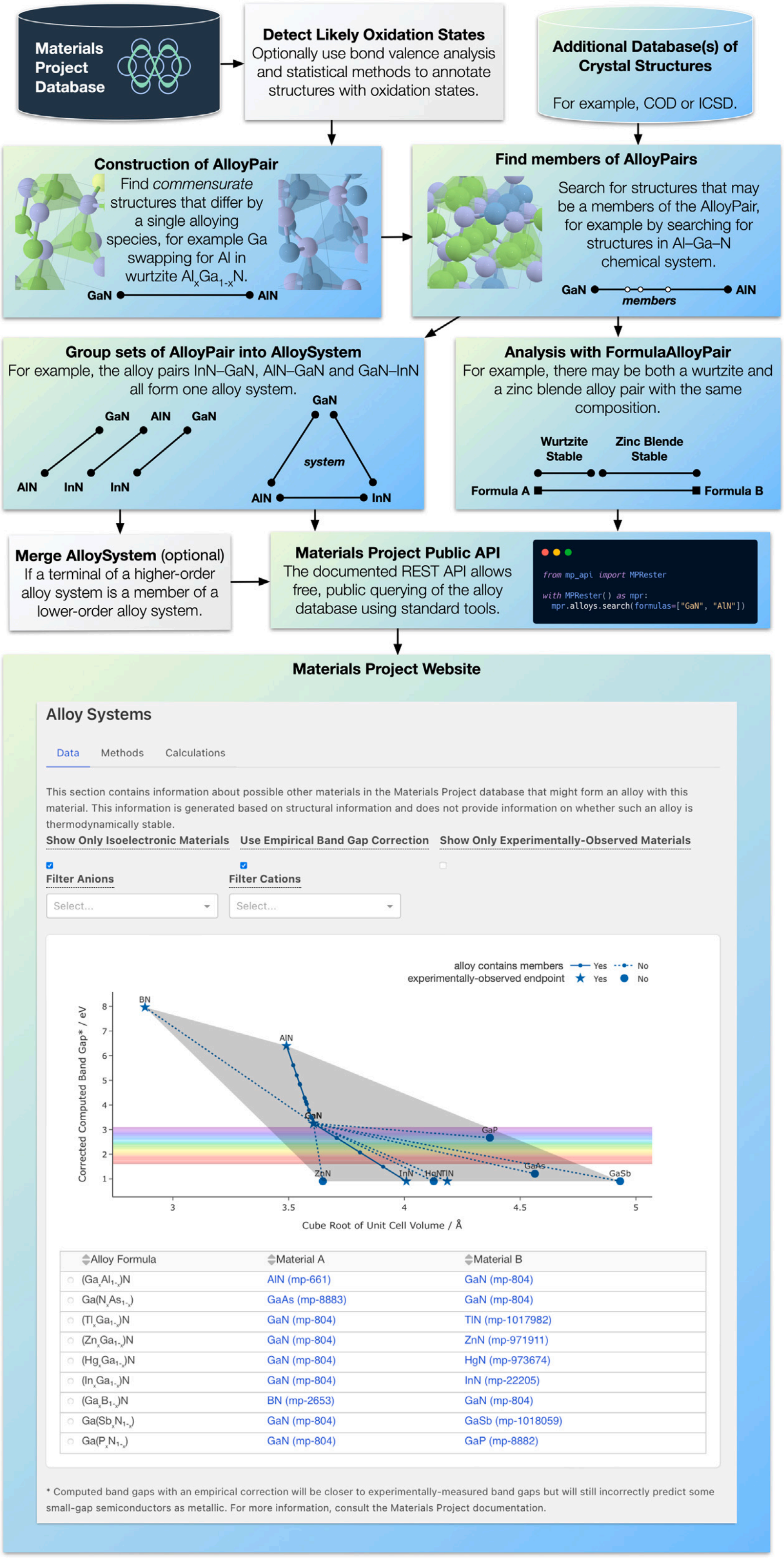

Figure 6. Alloys data processing pipeline

A flowchart showing the data processing pipeline outlined in the section "methodology," starting from a generic crystal structure database such as the MP, and ending with a publicly accessible API and Web site to explore the data. Wurtzite GaN is shown here as an example, as an alloy pair with InN and as an alloys system plotted on the MP Web site.

Methodology

We have created an open-source code, `pymatgen-analysis-alloys`, that allows the construction of an alloy database when provided with an input database containing crystal structures. As a demonstration, we apply this code to the MP database. The left side of Figure 6 depicts the data processing pipeline of the alloys database, as described here. This code is also used for the automatic generation of the plots shown in this manuscript, with only light additional editing performed for presentation.

The method outlined here does not require any prior knowledge of which materials might form alloys. While partial occupancies in, e.g., a Crystallographic Information File (.cif) indicates the possibility of alloying, this criterion only captures known systems and hence does not fully explore the possible alloy space. The challenge when constructing the database is in the data processing pipeline, and addressing combinatorial problems when large databases of hundreds of thousands of entries are used.

The method is as follows: for each crystal structure in the input database, designated as a potential endpoint, we find all other compounds that share its anonymous formula (e.g., ABC_2), and perform a pairwise comparison between all materials to detect commensurate structures using the `StructureMatcher` in `pymatgen`.[51] A pre-filter is performed that checks for detected space group, calculated with `spglib`,[52] using both tight and loose tolerances. This pre-filter is imposed with the logic that it is a necessary but not sufficient condition that two commensurate crystal structures will have the same space group. After a pair of crystal structures are identified as an endpoint, information is extracted such as the alloying species, including oxidation state, and whether the alloy is isoelectronic, and stored as an instance of an `AlloyPair` class. This definition of alloy does not consider alloys formed through interstitial alloying additions or other types of alloys. We apply the definition of commensurate from Holder et al.: "alloys between materials with commensurate lattices … have symmetries that are related by a displacive phase transformation" such that "structural distortions and atomic displacements lead to a continuous change of the lattice parameters and site positions without requiring atomic diffusion or rearrangement of the coordination environment."[26] Our alloy database will capture these commensurate examples if the displacements are small (such that the `StructureMatcher` algorithm considers them to be the same structure within a given tolerance) but not if displacements are large.

All `AlloyPair` entries contain structural properties, such as space group and primitive cell volume, but can be supplemented with additional properties. For this demonstration, supplemental properties are taken from the MP and include E_{hull}, E_G from the PBE functional from the MP, and electron and hole effective masses from Ricci et al.[40] (note that these are only computed for a subset of the MP database), but, in principle, this can be expanded to include any material property. Methods are provided to interpolate these properties using Vegard's law (assuming no bowing) for a given alloy content to allow for easier plotting and searching (see supplemental information).

Once a set of `AlloyPair` entries are constructed, they are grouped by chemical system and iterated over to search for potential members, defined by the `AlloyMember` class, using a similar approach. Determining whether a provided crystal structure can be assigned as a member to a given alloy pair requires both a exact match of stochiometry and, in addition, either an exact match of space group or a structure match. This stricter criteria reduces the total number of members that might be assigned and could be relaxed for some applications; for example, if a small off-stochiometry was allowable. Assigning membership allows a database query to reveal which alloys already have existing data available, and thus may be more experimentally accessible alloys when performing a screening.

A set of alloy pairs can be grouped together as an alloy system, defined by the class `AlloySystem`, using a network graph method whereby each edge in the graph is an alloy pair and connected subgraphs form the respective alloy systems. The code allows for alloy systems to be merged when a member of one system might be the endpoint of another system; for example, an alloy system with ternary endpoints where one endpoint is itself a member of a binary alloy pair.

Another useful grouping is the set of alloy pairs that all have the same set of endpoint formulae: these can be grouped together as a formula alloy pair, defined by the class `FormulaAlloyPair`. If formation enthalpies are known for the endpoints, this class is able to define regions where a given polymorph is stable according to a simple linear interpolation, and define alloy segments (class `AlloySegment`) that encode the critical alloy contents at which a phase transition may occur between two polymorphs. Furthermore, if any alloy members are known, including their formation enthalpies, these data will inform how accurate the simple linear interpolation may be. Examples of these can be seen in Figure 4.

For the example database generated in this work, we exclude compounds including H, He, noble gases, and heavy elements with atomic numbers greater than 83 (Bi), although all these entries are present in the underlying database, but we do not perform any further filtering based on chemistry and leave this as a capability for the user querying the database to decide exactly what chemical systems, maximum electronegativity differences, etc. are allowable for their specific design case.

The API to access and search the database is defined in the open-source `emmet` code. The user interface on the MP Web site is constructed using the open-source Crystal Toolkit Web framework. All open-source code described in this work is open to review and suggested edits by other researchers, and any contributions are welcomed by the authors.

SUPPLEMENTAL INFORMATION

Supplemental information can be found online at https://doi.org/10.1016/j.patter.2023.100723.

ACKNOWLEDGMENTS

This work was supported by the US Department of Energy, Office of Science, Office of Basic Energy Sciences, Materials Sciences and Engineering Division under contract no. DE-AC02-05-CH11231 (MP program KC23MP). R.W.R. acknowledges financial support from the UC Berkeley Chancellor's Fellowship and the National Science Foundation (NSF) Graduate Research Fellowship under grant no. DGE1106400 and DGE175814.

AUTHOR CONTRIBUTIONS

We highlight the author contributions to this study using the CRediT taxonomy. R.W.R., conceptualization, methodology, investigation, data curation, formal analysis, software, validation, visualization, writing – original draft, writing – review & editing; M.K.H., conceptualization, methodology, investigation, data curation, formal analysis, software, validation, visualization, writing – original draft, writing – review & editing; K.A.P., funding acquisition, project administration, resources, supervision, writing – review & editing.

DECLARATION OF INTERESTS

The authors declare no competing interests.

Received: July 14, 2022
Revised: December 19, 2022
Accepted: March 8, 2023
Published: April 7, 2023

REFERENCES

1. Mukai, T., Yamada, M., and Nakamura, S. (1999). Characteristics of ingan-based uv/blue/green/amber/red light-emitting diodes. Jpn. J. Appl. Phys. *38*, 3976.
2. Kinch, M.A. (2000). Fundamental physics of infrared detector materials. J. Electron. Mater. *29*, 809–817.
3. Anton, S.R., and Sodano, H.A. (2007). A review of power harvesting using piezoelectric materials (2003–2006). Smart Mater. Struct. *16*, R1–R21.
4. Dhere, N.G. (2006). Present status and future prospects of CIGSS thin film solar cells. Sol. Energy Mater. Sol. Cells *90*, 2181–2190.
5. Rubin, A.E. (1997). Mineralogy of meteorite groups. Meteoritics & Planetary Science *32*, 231–247.

CellPress
OPEN ACCESS

6. Zhu, H., Hautier, G., Aydemir, U., Gibbs, Z.M., Li, G., Bajaj, S., Pöhls, J.H., Broberg, D., Chen, W., Jain, A., et al. (2015). Computational and experimental investigation of TmAgTe 2and XYZ 2compounds, a new group of thermoelectric materials identified by first-principles high-throughput screening. J. Mater. Chem. C Mater. *3*, 10554–10565.
7. Chen, H., Hao, Q., Zivkovic, O., Hautier, G., Du, L.-S., Tang, Y., Hu, Y.-Y., Ma, X., Grey, C.P., and Ceder, G. (2013). Sidorenkite (Na 3MnPO 4CO 3): a new intercalation cathode material for na-ion batteries. Chem. Mater. *25*, 2777–2786.
8. Hautier, G., Miglio, A., Ceder, G., Rignanese, G.-M., and Gonze, X. (2013). Identification and design principles of low hole effective mass p-type transparent conducting oxides. Nat. Commun. *4*, 2292–2297.
9. Woods-Robinson, R., Broberg, D., Faghaninia, A., Jain, A., Dwaraknath, S.S., and Persson, K.A. (2018). Assessing high-throughput descriptors for prediction of transparent conductors. Chem. Mater. *30*, 8375–8389.
10. Li, R., Xie, L., Wang, W.Y., Liaw, P.K., and Zhang, Y. (2020). High-throughput calculations for high-entropy alloys: a brief review. Front. Mater. *7*.
11. Lederer, Y., Toher, C., Vecchio, K.S., and Curtarolo, S. (2018). The search for high entropy alloys: a high-throughput ab-initio approach. Acta Mater. *159*, 364–383.
12. Pitike, K.C., KC, S., Eisenbach, M., Bridges, C.A., and Cooper, V.R. (2020). Predicting the phase stability of multicomponent high-entropy compounds. Chem. Mater. *32*, 7507–7515.
13. Jiang, S., and Yang, K. (2021). Review of high-throughput computational design of heusler alloys. J. Alloys Compd. *867*, 158854.
14. Sanvito, S., Oses, C., Xue, J., Tiwari, A., Zic, M., Archer, T., Tozman, P., Venkatesan, M., Coey, M., and Curtarolo, S. (2017). Accelerated discovery of new magnets in the heusler alloy family. Sci. Adv. *3*, e1602241.
15. Garcia, C.A.C., Bocarsly, J.D., and Seshadri, R. (2020). Computational screening of magnetocaloric alloys. Phys. Rev. Mater. *4*, 024402.
16. Bhattacharya, S., and Madsen, G.K.H. (2015). High-throughput exploration of alloying as design strategy for thermoelectrics. Phys. Rev. B *92*, 085205.
17. Jain, A., Ong, S.P., Hautier, G., Chen, W., Richards, W.D., Dacek, S., Cholia, S., Gunter, D., Skinner, D., Ceder, G., and Persson, K.A. (2013). Commentary: the materials project: a materials genome approach to accelerating materials innovation. Apl. Mater. *1*, 011002.
18. Curtarolo, S., Setyawan, W., Wang, S., Xue, J., Yang, K., Taylor, R.H., Nelson, L.J., Hart, G.L., Sanvito, S., Buongiorno-Nardelli, M., et al. (2012). AFLOWLIB.ORG: a distributed materials properties repository from high-throughput ab initio calculations. Comput. Mater. Sci. *58*, 227–235.
19. Kirklin, S., Saal, J.E., Hegde, V.I., and Wolverton, C. (2016). High-throughput computational search for strengthening precipitates in alloys. Acta Mater. *102*, 125–135.
20. Bligaard, T., Jóhannesson, G.H., Ruban, A.V., Skriver, H.L., Jacobsen, K.W., and Nørskov, J.K. (2003). Pareto-optimal alloys. Appl. Phys. Lett. *83*, 4527–4529.
21. Yang, K., Oses, C., and Curtarolo, S. (2016). Modeling off-stoichiometry materials with a high-throughput ab-initio approach. Chem. Mater. *28*, 6484–6492.
22. Gubaev, K., Podryabinkin, E.V., Hart, G.L., and Shapeev, A.V. (2019). Accelerating high-throughput searches for new alloys with active learning of interatomic potentials. Comput. Mater. Sci. *156*, 148–156.
23. van de Walle, A., and Asta, M. (2019). High-throughput calculations in the context of alloy design. MRS Bull. *44*, 252–256.
24. Curtarolo, S., Hart, G.L.W., Nardelli, M.B., Mingo, N., Sanvito, S., and Levy, O. (2013). The high-throughput highway to computational materials design. Nat. Mater. *12*, 191–201.
25. Mizutani, U. (2012). Hume-rothery rules for structurally complex alloy phases. MRS Bull. *37*, 169.
26. Holder, A.M., Siol, S., Ndione, P.F., Peng, H., Deml, A.M., Matthews, B.E., Schelhas, L.T., Toney, M.F., Gordon, R.G., Tumas, W., et al. (2017). Novel phase diagram behavior and materials design in heterostructural semiconductor alloys. Sci. Adv. *3*, e1700270.
27. Bergerhoff, G., and Brown, I.D. (1987). Crystallographic databases/Allen FH et al.(hrsg.) (Chester, international union of crystallography).
28. Singh, J. (2007). Electronic and Optoelectronic Properties of Semiconductor Structures (Cambridge University Press).
29. Piprek, J. (2013). Semiconductor Optoelectronic Devices: Introduction to Physics and Simulation (Elsevier).
30. Woods-Robinson, R., Han, Y., Zhang, H., Ablekim, T., Khan, I., Persson, K.A., and Zakutayev, A. (2020). Wide band gap chalcogenide semiconductors. Chem. Rev. *120*, 4007–4055.
31. Morales-García, Á., Valero, R., and Illas, F. (2017). An empirical, yet practical way to predict the band gap in solids by using density functional band structure calculations. J. Phys. Chem. C *121*, 18862–18866.
32. Goede, O., Heimbrodt, W., Lamla, M., and Weinhold, V. (1988). Energy gap of mns. phys. stat. sol. *146*, K65–K69.
33. W.M. Haynes, ed. (2011). CRC Handbook of Chemistry and Physics, 92nd ed. (CRC Press).
34. Aykol, M., Dwaraknath, S.S., Sun, W., and Persson, K.A. (2018). Thermodynamic limit for synthesis of metastable inorganic materials. Sci. Adv. *4*, eaaq0148.
35. Curtarolo, S., Morgan, D., and Ceder, G. (2005). Accuracy of ab initio methods in predicting the crystal structures of metals: a review of 80 binary alloys. Calphad *29*, 163–211.
36. Sun, W., Dacek, S.T., Ong, S.P., Hautier, G., Jain, A., Richards, W.D., Gamst, A.C., Persson, K.A., and Ceder, G. (2016). The thermodynamic scale of inorganic crystalline metastability. Sci. Adv. *2*, e1600225.
37. Banerjee, A., and Chattopadhyay, K. (2005). Recent developments in the emerging field of crystalline p-type transparent conducting oxide thin films. Prog. Cryst. Growth Char. Mater. *50*, 52–105.
38. Sarmadian, N., Saniz, R., Partoens, B., and Lamoen, D. (2016). Easily doped p-type, low hole effective mass, transparent oxides. Sci. Rep. *6*, 20446–20449.
39. Varley, J.B., Samanta, A., and Lordi, V. (2017). Descriptor-based approach for the prediction of cation vacancy formation energies and transition levels. J. Phys. Chem. Lett. *8*, 5059–5063.
40. Ricci, F., Chen, W., Aydemir, U., Snyder, G.J., Rignanese, G.-M., Jain, A., and Hautier, G. (2017). An ab initio electronic transport database for inorganic materials. Sci. Data *4*, 170085.
41. Stevanović, V., Trottier, R., Musgrave, C., Therrien, F., Holder, A., and Graf, P. (2018). Predicting kinetics of polymorphic transformations from structure mapping and coordination analysis. Phys. Rev. Mater. *2*, 033802.
42. Therrien, F., Graf, P., and Stevanović, V. (2020). Matching crystal structures atom-to-atom. J. Chem. Phys. *152*, 074106.
43. Hautier, G., Fischer, C.C., Jain, A., Mueller, T., and Ceder, G. (2010). Finding nature's missing ternary oxide compounds using machine learning and density functional theory. Chem. Mater. *22*, 3762–3767.
44. Hautier, G., Fischer, C., Ehrlacher, V., Jain, A., and Ceder, G. (2011). Data mined ionic substitutions for the discovery of new compounds. Inorg. Chem. *50*, 656–663.
45. Laks, D.B., Ferreira, L., Froyen, S., and Zunger, A. (1992). Efficient cluster expansion for substitutional systems. Phys. Rev. B Condens. Matter *46*, 12587–12605.
46. Barroso-Luque, L., Yang, J.H., Xie, F., Chen, T., Kam, R.L., Jadidi, Z., Zhong, P., and Ceder, G. (2022). smol: a python package for cluster expansions and beyond. J. Open Source Softw. *7*, 4504.
47. Chen, A.-B., and Sher, A. (1995). Semiconductor Alloys: Physics and Materials Engineering (Springer Science & Business Media).
48. Zunger, A., Wei, S., Ferreira, L., and Bernard, J.E. (1990). Special quasirandom structures. Phys. Rev. Lett. *65*, 353–356.

49. Zakutayev, A., Wunder, N., Schwarting, M., Perkins, J.D., White, R., Munch, K., Tumas, W., and Phillips, C. (2018). An open experimental database for exploring inorganic materials. Sci. Data *5*, 180053–180112.

50. Talley, K.R., White, R., Wunder, N., Eash, M., Schwarting, M., Evenson, D., Perkins, J.D., Tumas, W., Munch, K., Phillips, C., and Zakutayev, A. (2021). Research data infrastructure for high-throughput experimental materials science. Patterns *2*, 100373.

51. Ong, S.P., Richards, W.D., Jain, A., Hautier, G., Kocher, M., Cholia, S., Gunter, D., Chevrier, V.L., Persson, K.A., and Ceder, G. (2013). Python materials genomics (pymatgen): a robust, open-source python library for materials analysis. Comput. Mater. Sci. *68*, 314–319.

52. Togo, A., and Tanaka, I. (2018). Spglib: a software library for crystal symmetry search. Preprint at arXiv. https://doi.org/10.48550/arXiv.1808.01590.

Patterns, Volume *4*

Supplemental information

A method to computationally screen for tunable properties of crystalline alloys

Rachel Woods-Robinson, Matthew K. Horton, and Kristin A. Persson

Supplemental Experimental Procedures

Open-source code

All code used in the preparation of this manuscript is open source. Where future developments require changes to the methods or algorithms described in this manuscript, these open source codes will contain the ground truth for how the alloy database is constructed.

The codes developed were:

- `pymatgen-analysis-alloys` An add-on package for the `pymatgen` code that contains the `AlloyPair`, `AlloyMember`, `AlloySystem` and `FormulaAlloyPair` classes and related logic.
- `emmet` This is an existing package containing information on how to build the databases used by the Materials Project. Code was added to `emmet-core` to define the database document schema and `emmet-builders` to define the scripts to construct the database in a scalable manner. Code was added to `emmet-api` to allow researchers to access the alloy database constructed in this work through the Materials Project.

At the time of writing, `pymatgen-analysis-alloys` is installable using the Python Package Index via `pip install pymatgen-analysis-alloys` and importable via `import pymatgen.analysis.alloys`. The main classes are located in `pymatgen.analysis.alloys.core` and are documented and unit tested. Readers are encouraged to refer to the code for any updates to this methodology subsequent to publication.

Unique Identifiers

This work uses a document-based database, namely MongoDB, which does not have an explicit schema. The database fields present will be derived based on the available attributes in the `AlloyPair` and other objects. The canonical reference for these attributes is the code itself.

Nevertheless, the use of a unique, primary key is essential for database management.

For `AlloyPair` this is an underscore-delimited string containing the unique identifiers of the endpoints from whatever input database is used. This implicitly assumes that an underscore is not used in the input databases's unique identifiers. For example, an `AlloyPair` consisting of materials mp-804 (GaN) and mp-661 (AlN) would have the unique identifier "mp-661_mp-804". The `AlloyPair` construction orders the endpoints deterministically, such that AlN will always be endpoint "A" and GaN will always be endpoint "B" regardless of the order of endpoints provided during construction.

For `AlloySystem`, the unique identifier is based on the first six digits of the MD5 hash of a sorted, underscore-delimited list of all unique identifiers of individual materials in that alloy system. This ensures that the identifier will change as additional members are added to the alloy system.

Database Building

Constructing the entire alloy database is CPU-bound and takes approximately one day on a 2.3 GHz 8-core Intel CPU. For Materials Project production purposes, this database build is typically parallelized across multiple nodes and "pleasingly parallel", since it can be parallelized across anonymous formula (for alloy pair and alloy system construction) and across chemical system (for alloy member construction) such that the total build time is greatly reduced.

Statistical analysis of interpolated half-space hull energies

In **Figure S1** we have performed a simple statistical analysis on a set of formula alloy pairs in which there is one or more values of x with at least two members (i.e. polymorphs) belonging to unique alloy pairs with unique endpoints. Rather than compare whether the interpolated energy of a member polymorph i (E_i^{interp}) precisely matches its DFT predicted from MP ($E_i^{\mathrm{hull,MP}}$), we instead have focused on whether the relative energy difference between two polymorphs is comparable (ΔE_{dif}). At the current build of the database, this sample set amounts to 379 values of x across 323 formula alloy pairs, and we perform statistical analysis on the two lowest energy polymorphs for each value of x. With this analysis, we show that approximately 64% of polymorphs are predicted using alloy pair interpolation to be within 25 meV of their MP orderings, and approximately 91% within 100 meV. Therefore a majority of cases are predicted to have the same polymorph ordering as in MP, although there are outliers and it is important to keep this in mind when using the alloy database.

With this statistical analysis we caution that we are not trying to prove a result, but rather provide insight into the distribution of data in the alloy database. The MP data here are formation energies of ordered approximations of alloys; these are not equivalent to the formation energies of the actual alloy (e.g., of a solid solution). MP has not done exhaustive enumerations of orderings, and it is unclear whether a given ordering is a useful approximation or not. We also note that there is bias in terms of which formula alloy pairs have members and which do not, inherited from the inherent bias in the MP database (e.g., oversampling of Li-containing systems due to battery applications; see Figure 4 and discussion), so these trends may not be

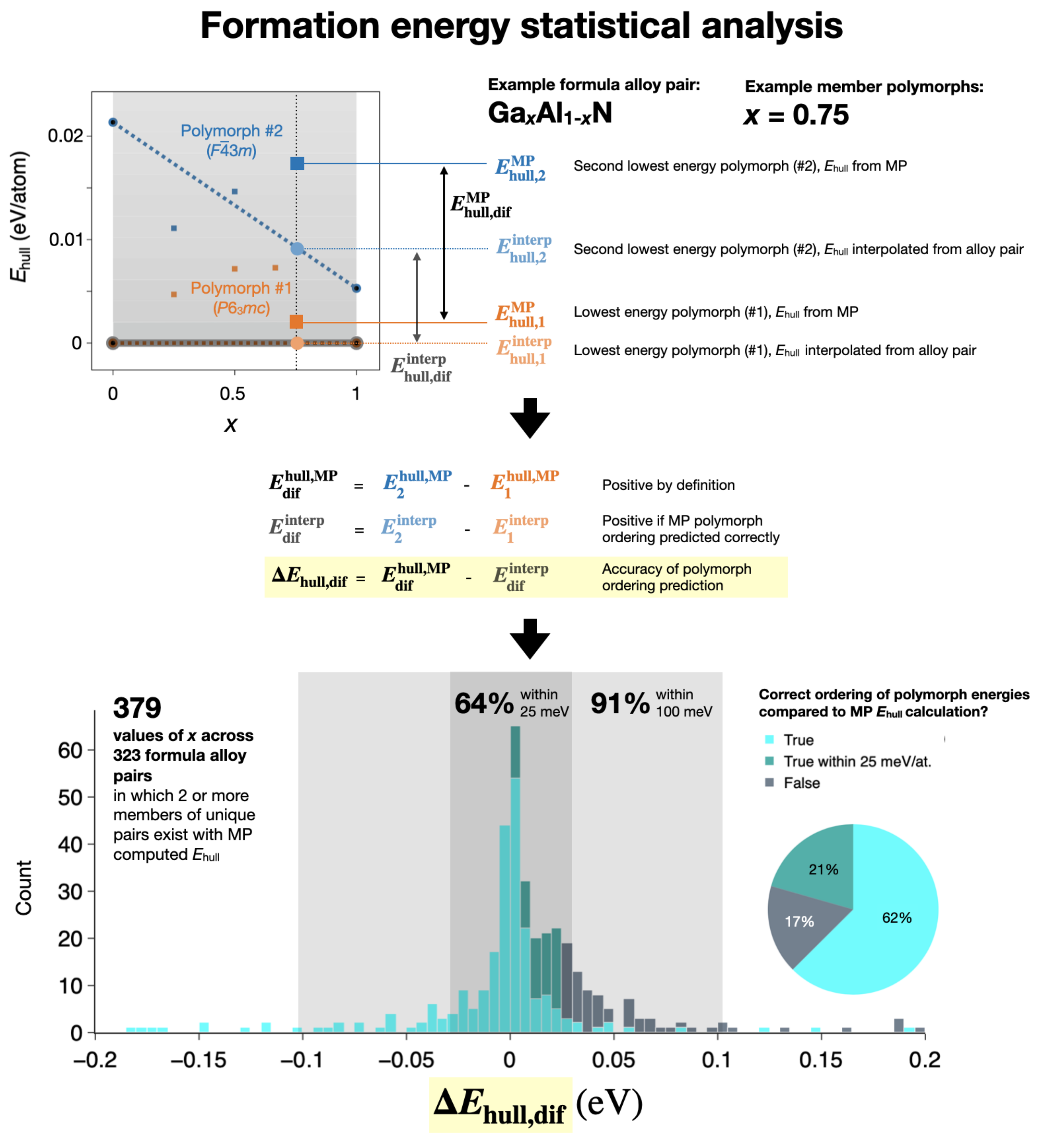

Figure S1: Half-space hull statistical analysis. A simple statistical analysis of interpolated polymorph ordering using the half-space hull framework. For reference, the formula alloy pair for $Ga_xAl_{1-x}N$ is shown (see Figure 4 in the manuscript, focusing on the two lowest-energy polymorphs at x=0.75. Square markers denote MP E_{hull} values, while circular markers denote interpolated values.

representative of all chemistries and structures. The trend here however is that, as the databases increase in size (e.g., more and more ordered approximations of different disordered materials are added), it will become more accurate.

Merging of AlloySystem

Consider there is an alloy system containing an endpoint with anonymous formula ABC. However, this endpoint ABC is also found to be a member of another alloy system (say, a system made up of the two endpoints, AB and AC). In this case, we can conclude the first alloy sys-

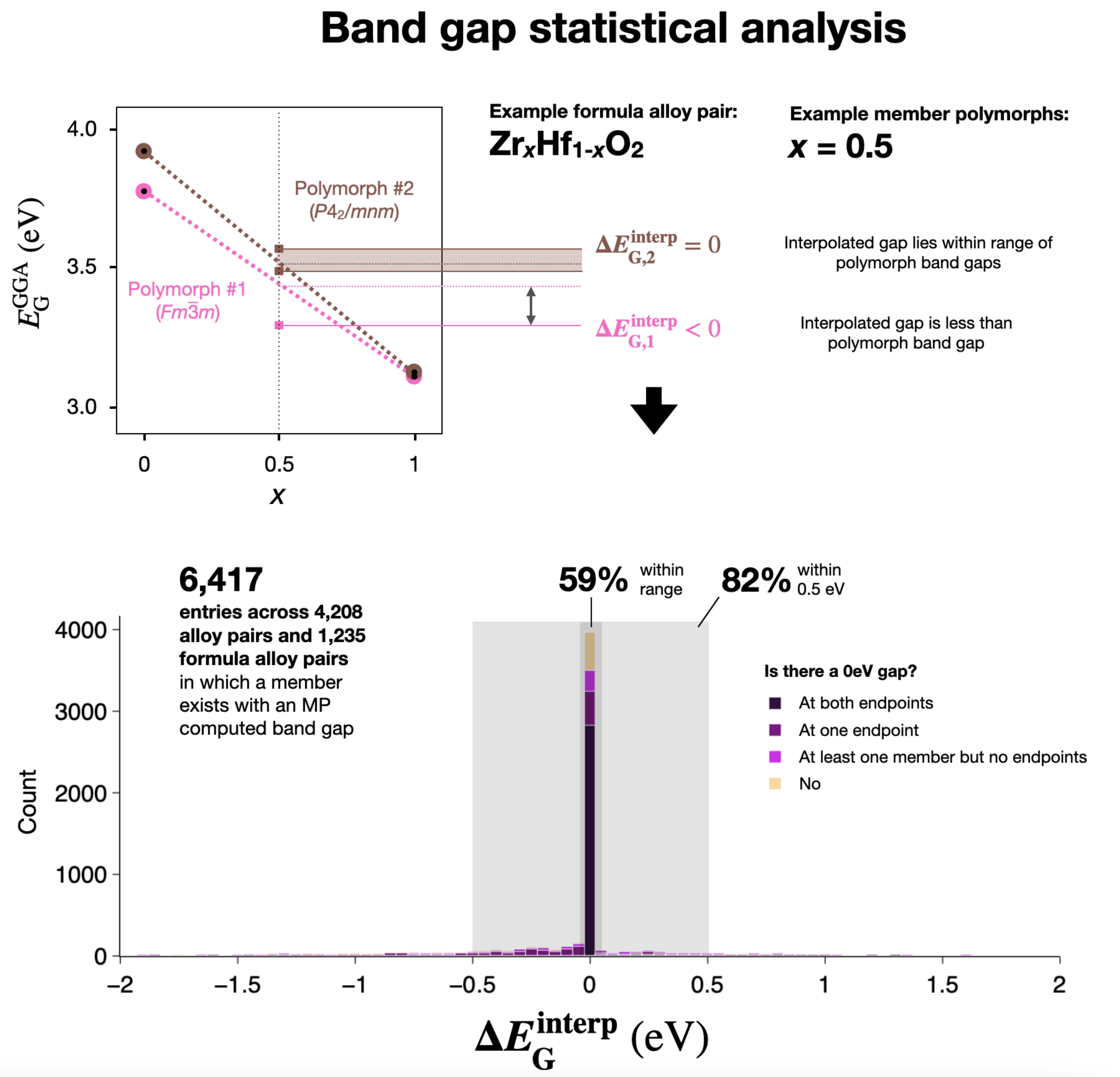

Figure S2: Band gap statistical analysis. A simple statistical analysis of interpolated band gap of alloy pair members, compared to their GGA band gaps on the MP database. For reference, the formula alloy pair for $Zr_xHf_{1\text{-}x}O_2$ is shown (see ??, focusing on the $P42/nmc$ and $Fm-3m$ polymorphs at x=0.5. Square markers denote MP E_{hull} values, while circular markers denote interpolated values.

tem can be subsumed into the second alloy system. This process of "merging" alloy systems is important to remove spurious alloy systems, but also presents a subtle problem since whether a material should be considered an alloy or, simply, a new stoichiometric compound is open to interpretation. For example, chalcopyrite is typically considered a compound in its own right, but under this lens would be seen as an alloy of two zincblende endpoints. Therefore, alloy system merging has not been performed on the database in this work, but has been fully implemented in the code and can be done manually on an as-needed basis.

Vegard's Law Approximations

In the manuscript, we assume Vegard's law applies with no bowing to construct Figure 5, and for properties a, E_G, and inverse effective mass (i.e. $\frac{1}{m_e^*}$ and $\frac{1}{m_h^*}$). This is a crude approximation for the purposes of providing a window for a given alloy in which properties might lie, *not* as a way to accurately estimate properties. Consensus in the literature is that Vegard's law is an approximation rather than a strict "law", but that it is valid as a predictor for lattice parameter for ideal solutions in which lattice parameters

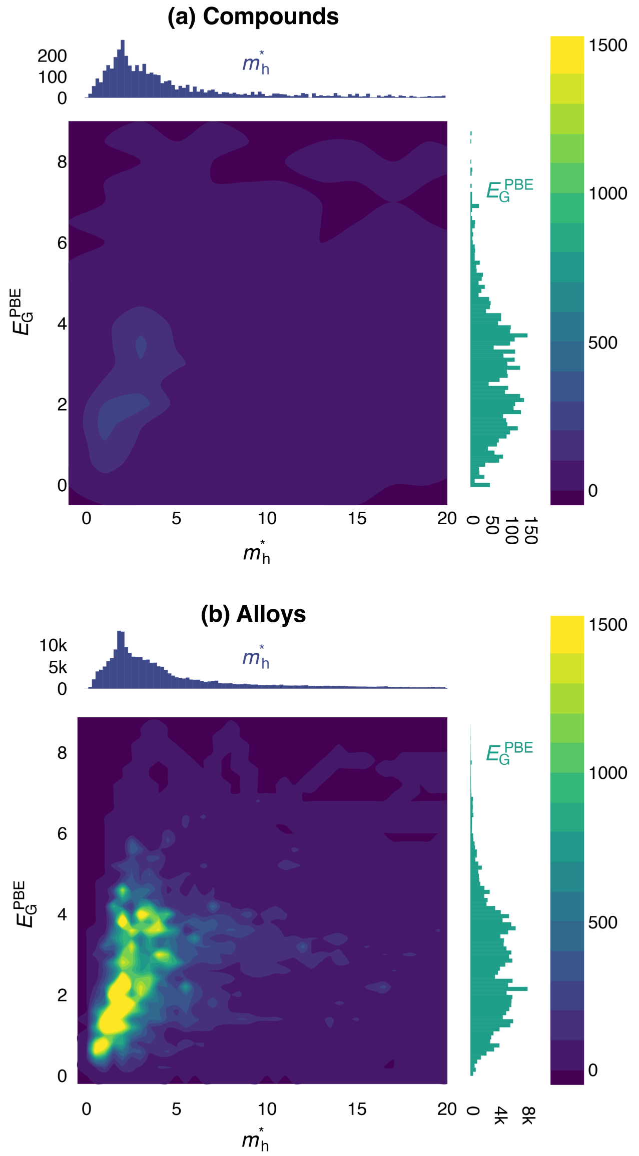

Figure S3: Density plot comparison. 2D density plots showing (a) the distribution of E_G and m^*_h when considering only stoichiometric alloy compounds ("endpoints") and (b) an approximate distribution of E_G and m^*_h including the intermediate alloy compositions, illustrating the different between a discrete and practically continuous distribution of properties.

of the end-points differ by less than 5 %.[1] In **Figure S2** we have performed a simple statistical analysis of Vegard's law interpolated band gaps of alloy pair members, compared to their GGA band gaps on the MP database. At the current build of the database, this sample set amounts to 6,417 entries across 4,208 alloy pairs and 1,235 formula alloy pairs in which a member exists with an MP computed band gap. With this analysis, we show that approximately 59% of interpolated gaps lie within range of MP polymorph band gaps, and approximately 82% lie within 0.5 eV of the MP gap(s).

The literature commonly applies Vegard's law for alloys to estimate a and E_G. In comparison, there is less consensus across the literature about whether Vegard's law is appropriate for m^* and whether bowing is pronounced; this is likely dependent on the specific characteristics of the electronic band structure for the alloy endpoints. According to Piprek[2] and Singh[3], Vegard's law is appropriate for inverse effective mass with the latter providing a derivation. According to Piprek "bowing is not pronounced for the effective mass of most alloys," as compared to stronger bowing for band gap. Here, "most alloys" is likely referring to III-V materials, since these are the dominant class of alloys studied — and for III-Vs, Vegard's law is used in the literature to estimate effective mass e.g. for (AlGaIn)N alloys.[4] Several materials properties, such as the piezoelectric response, are highly nonlinear as a function of alloy content and use of Vegard's law is not appropriate in these cases. Therefore, care must be taken in the choice of which properties associated with a material are included in the database. Moreover, the end user of the database has the ability to incorporate additional properties of the endpoints in whatever manner is most appropriate for their specific property or application.

Alloys increase parameter space

To graphically illustrate how including alloys increases parameter space, **Figure S3** depicts a 2D contour plot of two representative material properties — m^*_h versus PBE E_G (see Methodology) — for (a) compounds in the MP database, i.e. endpoints only, in comparison to (b) candidate alloy materials with steps of $\delta x = 0.01$ in an alloy pair $A_xB_{1\text{-}x}$ and assuming Vegard's law with no bowing for E_G and $1/m^*_h$.[3] The histograms above and to the right of each diagram depict the distribution for each individual parameter. Note that this is only an illustration to show the expanded property space accessible when considering alloys, and cannot be not a quantitative comparison since the choice of δ is arbitrary.

[1] Jacob, K., Raj, S., and Rannesh, L. (2007). Vegard's law: a fundamental relation or an approximation? *International Journal of Materials Research*, 98(9):776–779.

[2] Piprek, J. (2013). *Semiconductor optoelectronic devices: introduction to physics and simulation.* Elsevier.

[3] Singh, J. (2007). *Electronic and optoelectronic properties of semiconductor structures.* Cambridge University Press.

[4] Yang, H., Song, T., Liang, X., and Zhao, G. (2015). First-principle study of the electronic band structure and the effective mass of the ternary alloy gaxin1-xp. In *Journal of Physics: Conference Series*, volume 574, page 012048. IOP Publishing.
